# Extracellular vesicles derived from CD4^+^ T cells carry DGKK to promote sepsis-induced lung injury by regulating oxidative stress and inflammation

**DOI:** 10.1186/s11658-023-00435-y

**Published:** 2023-03-23

**Authors:** Guo-wei Tu, Yi Zhang, Jie-fei Ma, Jun-yi Hou, Guang-wei Hao, Ying Su, Jing-chao Luo, Lulu Sheng, Zhe Luo

**Affiliations:** 1 Cardiac Intensive Care Center, Zhongshan Hospital, Fudan University, Shanghai, China; 2 Biomedical Research Center, Institute for Clinical Sciences, Zhongshan Hospital, Fudan University, Shanghai, China; 3 Department of Critical Care Medicine, Zhongshan Hospital (Xiamen), Fudan University, Xiamen, China; 4 Department of Emergency Medicine, Shanghai Jiao Tong University Affiliated Sixth People’s Hospital, Shanghai, China; 5 Shanghai Key Laboratory of Lung Inflammation and Injury, Shanghai, China; 6 Department of Critical Care Medicine, Shanghai Xuhui Central Hospital, Zhongshan-Xuhui Hospital, Fudan University, Shanghai, China

**Keywords:** Sepsis-induced lung injury, DGKK, Extracellular vesicles, Oxidative stress, Inflammation

## Abstract

**Background:**

Sepsis is an abnormal immune response after infection, wherein the lung is the most susceptible organ to fail, leading to acute lung injury. To overcome the limitations of current therapeutic strategies and develop more specific treatment, the inflammatory process, in which T cell-derived extracellular vesicles (EVs) play a central role, should be explored deeply.

**Methods:**

Liquid chromatography–tandem mass spectrometry was performed for serum EV protein profiling. The serum diacylglycerol kinase kappa (DGKK) and endotoxin contents of patients with sepsis-induced lung injury were measured. Apoptosis, oxidative stress, and inflammation in A549 cells, bronchoalveolar lavage fluid, and lung tissues of mice were measured by flow cytometry, biochemical analysis, enzyme-linked immunosorbent assay, quantitative real-time polymerase chain reaction, and western blot.

**Results:**

DGKK, the key regulator of the diacylglycerol (DAG)/protein kinase C (PKC) pathway, exhibited elevated expression in serum EVs of patients with sepsis-induced lung injury and showed strong correlation with sepsis severity and disease progression. DGKK was expressed in CD4^+^ T cells under regulation of the NF-κB pathway and delivered by EVs to target cells, including alveolar epithelial cells. EVs produced by CD4^+^ T lymphocytes exerted toxic effects on A549 cells to induce apoptotic cell death, oxidative cell damage, and inflammation. In mice with sepsis induced by cecal ligation and puncture, EVs derived from CD4^+^ T cells also promoted tissue damage, oxidative stress, and inflammation in the lungs. These toxic effects of T cell-derived EVs were attenuated by the inhibition of PKC and NOX4, the downstream effectors of DGKK and DAG.

**Conclusions:**

This approach established the mechanism that T-cell-derived EVs carrying DGKK triggered alveolar epithelial cell apoptosis, oxidative stress, inflammation, and tissue damage in sepsis-induced lung injury through the DAG/PKC/NOX4 pathway. Thus, T-cell-derived EVs and the elevated distribution of DGKK should be further investigated to develop therapeutic strategies for sepsis-induced lung injury.

**Supplementary Information:**

The online version contains supplementary material available at 10.1186/s11658-023-00435-y.

## Background

Sepsis is an abnormal immune response after infection, wherein the lung is the most susceptible organ to fail, leading to acute lung injury (ALI) and progression of acute respiratory distress syndrome (ARDS) [1]. Sepsis-induced ALI is characterized by the enhanced permeability of cells in pulmonary capillary and alveolar epithelia, which might be caused by stepwise inflammation in airspaces and lung parenchyma [1]. Patients with ARDS often present with large amounts of proinflammatory and neutrophil chemotactic cytokines in bronchoalveolar lavage fluid (BALF) [2]. Together with these cytokines, various kinds of immune cells, particularly alveolar macrophages, neutrophils, regulatory T cells, and Th17 cells, are involved in the pathogenesis of ARDS [2].

Oxidative stress is frequently associated with ALI/ARDS [3]. Reactive oxygen species (ROS) are elevated under ALI/ARDS and accelerate tissue damage through multiple mechanisms, such as DNA damage, peroxidation of lipid molecules, oxidation of proteins that changes protein activity, and activation of transcription factors (including inflammatory NF-κB) that trigger the expression of proinflammatory genes [4]. The normal lung tissue produces various types of endogenous antioxidants, including superoxide dismutase (SOD). However, these antioxidants are not sufficient to protect the lungs from prolonged oxidative stress in ALI/ARDS [4].

Extracellular vesicles (EVs) originate from endosomes, with diameters ranging between 30 and 150 nm; they are frequently detected in urine, blood, and cerebrospinal fluid [5]. EVs carry cargo containing multiple cellular components (DNA, lipids, proteins), transposable elements, and RNA (coding and noncoding) [6]. They play an indispensable role in chronic inflammatory lung diseases, including chronic obstructive pulmonary disease (COPD) [7] and asthma [8, 9]. In the human body, several types of cells secrete EVs, including T lymphocytes [10, 11]. T-lymphocyte-derived EVs impair the function of salivary gland epithelial cells by inhibiting Ca^2+^ flux, cAMP production, and protein secretion [12] and trigger β cell apoptosis [13]. EVs derived from CD4^+^ T cells increase the antitumor response of CD8^+^ T cells by enhancing their proliferation and activity [14], induce NOX4-dependent oxidative stress in cardiac microvascular endothelial cells [15], and promote the proliferation, migration, and differentiation of cardiac fibroblasts to improve cardiac remodeling following myocardial infarction [16]. CD4^+^ T cells are important lymphocytes that play crucial roles in modulating immune responses during sepsis and lung injury. However, the roles of CD4^+^ T-cell-derived EVs in sepsis-induced lung injury remain unknown.

Diacylglycerol kinase (DGK) phosphorylates diacylglycerol (DAG) to produce phosphatidic acid (PA). DAG and PA play essential roles as critical secondary messengers in cell signaling. DAG activates the Rho/protein kinase C (PKC) pathways, including cPKC, nPKC, and aPKC pathways, whereas PA controls the Raf and mTOR pathways [17]. In mammals, DGK contains ten isozymes (α–κ) and regulates various important functions in cellular biochemistry and physiology [18]. DGK alpha regulates the metastasis of non-small lung cancer [19] and airway contraction in asthma pathogenesis [20]. DGK zeta supports inflammatory reaction and hyperresponsiveness in allergic airways [21]. DGK kappa (DGKK) participates in fragile X syndrome [22], and its nucleotide variants are associated with hypospadias [23]. Although DAG, PKC, and NOX4 are involved in sepsis or ALI [24, 25], the roles of DGK in sepsis-induced lung injury remain unknown.

To elucidate the role of EVs in sepsis-induced lung injury, the present study analyzed the serum EV protein profile of patients with sepsis-induced lung injury. DGKK, the key regulator of the DAG/PKC pathway, exhibited elevated expression in serum EVs of patients and showed strong correlation with sepsis severity and progression. EVs derived from lipopolysaccharide (LPS)-treated CD4^+^ T cells carrying DGKK induced oxidative stress and inflammation in alveolar epithelial A549 cells and sepsis-induced mice through PKC and NOX4, the downstream effectors of DGKK and DAG. Therefore, this approach established the mechanism that T-cell-derived EVs exerted toxic effects in sepsis-induced lung injury through the DGKK/DAG/PKC/NOX4 pathway. Thus, T-cell-derived EVs and the elevated distribution of DGKK should be further investigated to develop therapeutic strategies for sepsis-induced lung injury.

## Materials and methods

### Criteria for subject selection and collection of clinical samples

Forty patients with sepsis with lung injury, as defined by the criteria of the North American European Consensus Conference, were enrolled in this study [26]. The exclusion criteria were as follows: (1) patients who had cancer or hematological malignancy, (2) cases that were complicated with autoimmune disease, and (3) patients who were pregnant or breastfeeding. In addition, 20 healthy subjects who had no abnormalities in medical examination were included as healthy controls. The exclusion criteria for healthy controls were similar to those of patients with sepsis. Patients with sepsis with lung injury were paired with the healthy control group in terms of gender, age, and other systemic diseases. The morning fasting venous blood of patients with sepsis was collected within 24 h after admission and centrifuged at 3000 rpm for 15 min. The supernatant was frozen in a refrigerator at −80 °C to avoid repeated freezing and thawing. Then, it was uniformly subjected to EV extraction. The protocols of the present study were approved by the hospital’s ethics committee. All participants provided informed consent to be involved in this study.

### Cell culture and treatment

A549 cells (ATCC CCL-185, USA) were cultured in Dulbecco’s modified Eagle medium (DMEM) with 10% fetal bovine serum (FBS; Gibco, USA) and penicillin–streptomycin mixture (100 × dilution; Solarbio Science & Technology, China) in 5% CO_2_ at 37 °C. Peripheral CD4^+^ T cells isolated from healthy control subjects were purified using a CD4^+^ T cell Isolation Kit (Miltenyi, China) and activated in vitro with 2 μg/mL plate‐bound anti‐CD3/CD28 antibodies (eBioscience, San Diego, CA, USA). The isolated CD4^+^ T cells (1.5 × 10^6^ cells/mL) were cultivated in a 24‐well plate and stimulated with 2 μg/mL plate‐bound anti‐CD3/CD28 antibodies at 37 °C and 5% CO_2_ for 24 h in FBS‐free RPMI-1640. Then, they were treated with or without 10 μg/mL LPS for 24 h, and the conditioned medium was collected for EV isolation. A549 cells cultured in FBS‐free DMEM were treated with 100 μg/mL EVs isolated from the above-mentioned CD4^+^ T cells, followed by 2 nM LXS-196 (Selleck Chemicals LLC, Houston, TX, USA) or 5 μM GLX351322 (MedChemExpress, Monmouth Junction, NJ, USA) for 24 h.

### Isolation and identification of EVs

The EVs derived from the above-mentioned CD4^+^ T cells and serum were isolated and purified in accordance with a previously described method with some modifications [27]. Briefly, the serum samples, which were thawed in a water bath at 25 °C and placed on ice, and conditioned medium of CD4^+^ T cells with or without LPS treatment, were harvested and centrifuged at 4 °C at 2000 × *g* for 10 min. Then, the supernatant was taken. Centrifugation was performed at 10,000 × *g* at 4 °C for 30 min, and the supernatant was taken. The samples were transferred to an ultra-high-speed centrifuge tube (Backman Avanti J-30i, Shanghai, China) at 4 °C and centrifuged at 110,000 × *g* for 75 min. Then, the supernatant was discarded. The precipitates were suspended in 1 mL of 1 × phosphate-buffered saline (PBS), diluted with 1 × PBS, and filtered with 0.22 μm membrane after suspension. Next, the samples were transferred to an ultra-high-speed centrifuge tube at 4 °C and centrifuged at 110,000 × *g* for 75 min, and the supernatant was discarded. The precipitation was resuspended with the corresponding 1 × PBS, separated, and stored at −80 °C. The protein content of the concentrated EVs was determined using a BCA protein assay kit. The protein abundance of CD9 (Abcam, Waltham, MA, USA; ab236630), CD81 (Abcam; ab109201), TSG101 (Abcam; ab133586), and GM130 (Abcam; ab52649) was determined by western blot.

### Liquid chromatography–tandem mass spectrometry (LC–MS/MS)

The serum EVs of three patients with sepsis-induced lung injury and three healthy control subjects were selected for LC–MS/MS analysis. The samples were separated by the Easy-nLC system (with an nL flow rate) and analyzed with the Q Exactive Plus MS system [28]. The proteins were recognized and quantified on the basis of the Uniprot_HomoSapiens_20367_20200226 database. MaxQuant software (1.5.5.1) was employed for database searching, and the LFQ algorithm was used for quantitatively analyzing the peptides identified [29].

### Transmission electron microscopy (TEM)

The purified EVs were fixed with 4% paraformaldehyde (Electron Microscopy Science, USA) in PBS for 20 min at room temperature. Then, the fixed EVs were loaded on the carbon-coated grid and fixed with 4% paraformaldehyde for 30 s. The grid was examined using a TEM (JEM-1400plus, Japan) according to the manufacturer’s instructions.

### Nanoparticle tracking analysis (NTA)

Exosomes were diluted to achieve 140–200 particles per frame. ZetaView inspection instrument (Particle Metrix, Meerbusch, Germany) was used to determine the size and number of exosome particles. After loading the exosome samples into the sample chamber, the manufacturer’s settings for nanospheres were set, and the data were captured and calculated by the NTA software (ZetaView 8.04.02).

### EV uptake

A549 cells were used to specifically label EVs with PKH67 Green Fluorescent Probe (Sigma–Aldrich, USA). The signal was visualized as the EVs were taken up by A549 cells. Briefly, the EVs were initially diluted with the kit’s component Diluent C and then carefully incubated with PKH67 Green Fluorescent Probe at 25 °C for 5 min. Next, sterile FBS was added, and the mixture was allowed to sit for 1 min for sufficient staining. Then, the mixture was transferred, washed with basal medium, and centrifuged at the highest speed at 4 °C for about 75 min. The labeled EVs remained at the bottom of the tubes after discarding the upper layer supernatant. Then, they were resuspended by basal medium. Approximately 1 μg of labeled EVs was taken and added into A549 cells on a round-shaped coverslip in a 24-well plate. Then, they were incubated in standard condition (5% CO_2_, 37 °C) for 16 h or overnight. Afterward, the cells were washed and fixed with 4% paraformaldehyde for 10 min. The slides were then mounted with antifluorescence quenching agent together with DAPI.

### Animals and study design

Wild-type, male, C57BL/6 mice aged 6–8 weeks were obtained from Sippr-BK Laboratory Animal, Shanghai, China. All animal experiments were conducted according to the rules approved by the hospital’s ethics committee. The model with sepsis-induced lung injury (model group) was developed by performing cecal ligation and puncture (CLP). The animals were anesthetized using 1% pentobarbital sodium through intraperitoneal injection. Then, a midline incision (4 mm) was made to expose the cecum, which was later sutured with 3–0 silk suture, followed by a double “through and through” perforation with a 20-gauge needle about 5 mm from the ligature. The injured cecum was then repositioned and sterilized. After these careful operations for inducing injury, all animals were resuscitated. All animals were subcutaneously injected with 0.9% NaCl for resuscitation.

The mice were randomized into the CLP, CLP + SE (EVs isolated from healthy subjects), CLP + SSE group (EVs isolated from patients with sepsis-induced lung injury), CLP + TE (EVs isolated from CD4^+^ T cells isolated from healthy subjects), CLP + LTE (EVs isolated from CD4^+^ T cells isolated from healthy subjects treated with 10 μg/mL LPS), CLP + LTE + vehicle, CLP + LTE + LXS-196, CLP + LTE + GLX351322, CLP + shNC, and CLP + shDgkk groups. To examine the influence of EVs in lung injury and the survival of septic mice, 200 μL of EVs was intravenously injected into mice via the tail vein at 4 h after CLP surgery in accordance with previously described methods with some modifications [30, 31]. To evaluate the function of PKC/NOX4 on EV function, the PKC inhibitor LXS-196 (5 mg/kg/day; Selleck Chemicals, USA) and NOX4 inhibitor GLX351322 (5 mg/kg/day; Medchemexpress, USA) were injected intraperitoneally into mice at 1 h after CLP surgery. *Dgkk* silenced mice were established by intravenously injecting 1 × 10^8^ pfu/mL of shRNA-Dgkk adenovirus (the dose preoptimized before experiments) via the tail vein at 1 h after CLP. The mice were euthanized 24 h later. BALF was collected, and lung tissue was harvested and fixed in 4% paraformaldehyde for 24 h for hematoxylin and eosin (H and E) staining. The severity of histological injury in different groups was assessed by using a scoring system as previously described [32].

### Adenovirus production

A recombinant pShuttle-H1 adenovirus vector, containing shRNA-Dgkk targeting the mouse *Dgkk* gene (shDgkk), and control pShuttle-H1 adenovirus, containing nonspecific shRNA sequence (shNC), were constructed by Novobio Biotech (Shanghai, China). The sequence of shRNA is as follows: shDgkk, 5′-GGA ATG CAC TAC TGG TAT T-3′. Scramble shRNA (shNC, 5′-GAG CAT GTA GCA CTA TGT T-3′) was used as negative control. The packaging, purification, and titration of the above-mentioned adenovirus were carefully performed [33].

### Cell transfection

siRNAs targeting human NOX4 were synthesized by GenePharma Corporation (Shanghai, China) and transfected into A549 cells. The sequences of siRNA are as follows: siNOX4-1 5′-GGG CUA GGA UUG UGU CUA ATT-3′, siNOX4-2 5′- CAG UGA AGA CUU UGU UGA ATT-3′, and siNOX4-3 5′-GCA AGA CCU GGU CAG UAU ATT-3′. Scramble siRNA (siNC 5′-UUC UCC GAA CGU GUC ACG UTT-3′) was used as negative control.

### Enzyme-linked immunosorbent assay (ELISA)

About 100 μL of EVs was resuspended in radioimmunoprecipitation assay (RIPA) buffer and added with 100 μL of protease inhibitor mix (freshly added before use). The DGKK levels in serum EVs were measured using the DGKK ELISA Kit (4A Biotech Co. Ltd., Beijing, China). The levels of tumor necrosis factor (TNF)-α, interleukin (IL)-6, and IL-1β in serum, BALF, or A549 cells were measured using an assay kit (Nanjing Jiancheng, China) following the manufacturer’s protocols. DAG levels and PKC activity in mouse lung tissues or A549 cells were measured using the Diacylglycerol Assay Kit (Abcam, USA) and PKC Kinase Activity Kit (Enzo Life Sciences, USA), respectively.

### Measurement of organ injury markers

Plasma levels of alanine aminotransferase (ALT), aspartate aminotransferase (AST), and lactate dehydrogenase (LDH) were measured using commercial assay kits (Pointe Scientific, Lincoln Park, MI, USA) according to the manufacturer’s instructions.

### Measurement of ROS, malondialdehyde (MDA), SOD, glutathione peroxidase (GPX), and endotoxin

The levels of MDA, ROS, and SOD in human serum samples, mouse lung tissues, or A549 cells were measured using an assay kit (Nanjing Jiancheng, China) following the manufacturer’s protocols. GPX levels in human serum samples, mouse lung tissues, or A549 cells were measured using the Micro GPX Kit (Beijing Solarbio, China). Endotoxin levels in human serum samples were measured using the ToxinSensor LAL Endotoxin Kit (GenScript, NJ, USA).

### Apoptosis analysis using flow cytometry

A549 cells were seeded at a density of 3 × 10^5^ cells per well. Next, they were added with 5 μL of Annexin V-FITC solution and incubated at 4 °C for 15 min. Then, they were added with 5 μL of propidium iodide (PI) and incubated for 15 min. Cell apoptosis was examined on an Accuri C6 flow cytometer (BD Biosciences, USA).

### Detection of intracellular ROS levels

ROS production in A549 cells was detected by measuring 2′,7′-dichlorofluorescein diacetate (DCFH-DA) with a flow cytometer following the instructions of the Reactive Oxygen Species Assay Kit (Beyotime Biotech, China).

### Quantitative real-time polymerase chain reaction (RT-qPCR)

Total messenger RNA was extracted from A549 and CD4^+^ T cells using TRIzol reagent (Thermo Fisher, USA). mRNA was then reverse-transcribed to produce cDNA using the RevertAid™ cDNA Synthesis Kit (Thermo Fisher, USA). RT-qPCR was conducted using SYBR Mix (Thermo Fisher, USA). The primers for PCR were designed by Invitrogen software, and their sequences are listed in Table 1. RT-qPCR was conducted on the 7300 Real-Time PCR System (Applied Biosystems, USA). GAPDH was used as internal control.

**Table 1:** Primer sequences used in the study

Gene	Forward/reverse	Sequence (5′–3′)
DGKK	Forward	AAGAAACAGTCAGGGTCAAC
	Reverse	AGGATGGAATGGTGCTAATG
NOX1	Forward	ATAGCAGAAGCCGACAGG
	Reverse	CCACCAATGCCGTGAATC
NOX2	Forward	TAAGATAGCGGTTGATGGG
	Reverse	CAGATTGGTGGCGTTATTG
NOX3	Forward	TTGGCGTGTTCTTCTGTG
	Reverse	TCCTGGTGGAGTTCTTTG
NOX4	Forward	GACTTGGCTTTGGATTTCTG
	Reverse	TCTGAGGGATGACTTATGAC
NOX5	Forward	TGCACTGGGCAAGAATGAC
	Reverse	AGCAGCCACTTTCTGGAAC
DUOX1	Forward	AAGTCTCGCCTTATGTTC
	Reverse	ATCTTCCCATGTCAGTTC
DUOX2	Forward	GAACATCGCTGTGTATGAGTG
	Reverse	TTCTCCCGAATCCAGTAGTTG
IL6	Forward	GCACCTCAGATTGTTGTTG
	Reverse	AGTGTCCTAACGCTCATAC
IL1B	Forward	ATCAGCCAGGACAGTCAG
	Reverse	GAAGCGGTTGCTCATCAG
TNF	Forward	GGTATGAGCCCATCTATCTG
	Reverse	AGGGCAATGATCCCAAAG
GAPDH	Forward	GGAGCGAGATCCCTCCAAAAT
	Reverse	GGCTGTTGTCATACTTCTCATGG
Dgkk	Forward	GGAATTACTGCAACGCTCTTAC
	Forward	AACCAAAGATTGCCACAACC
Il6	Forward	TGGAGCCCACCAAGAACGATAG
	Reverse	TGTCACCAGCATCAGTCCCAAG
Il1b	Forward	GCATCCAGCTTCAAATCTC
	Reverse	ACACCAGCAGGTTATCATC
Tnf	Forward	GTGCTCAGAGCTTTCAAC
	Reverse	ACTCTCCCTTTGCAGAAC
Gapdh	Forward	CTGCCCAGAACATCATCC
	Reverse	CTCAGATGCCTGCTTCAC

### Western blot analysis

Lysate of A549 and CD4^+^ T cells was quickly generated in precooled RIPA buffer. NE-PER Extraction Reagents (Thermo Fisher Scientific) were used to prepare the cytosolic and nuclear fractions according to the protocol provided by the manufacturer along with the reagent. The protein concentration was measured using the BCA kit (Thermo Fisher Scientific) according to the manufacturer’s protocols. The protein extract was resolved in 10–15% SDS-PAGE gel and transferred and electroblotted onto nitrocellulose membrane (Millipore). The membrane was briefly washed and blocked through incubation with 5% non-fat milk. Next, it was added with primary antibodies against DGKK (Abcam; ab103681), TLR4 (Abcam; ab13867), NOX4 (Abcam; ab154244), NF-κBp65 (Cell Signaling Technology, Danvers, MA, USA; #8242), H3 (Cell Signaling Technology; #4499), or GAPDH (Cell Signaling #5174) at 4 °C for 16 h. The secondary antibodies conjugated with horseradish peroxidase (Cell Signaling Technology) were used for chemiluminescent signal determination. Immunoreactive signals were revealed by using the ECL chromogenic substrate kit (Bio-Rad, Hercules, USA).

### Luciferase reporter assay

The DGKK promoter reporter plasmid, with either wild-type or mutant sequence, was constructed by cloning the PCR-amplified promoter cDNA into the pGL3-Enhancer firefly luciferase reporter plasmid. CD4^+^ T cells treated with 10 μg/mL LPS and the NF-κB inhibitor QNZ (10 nM; Santa Cruz Biotech, USA) were seeded in flat-bottom 24-well plates and transfected transiently with reporter plasmid with DGKK promoter (each for 40 ng, wild type or mutant). To normalize the reporter read-out with the transfection efficiency for each individual sample, the cells were co-transfected with pRL-TK (50 ng), so that the renilla luciferase was encoded. After 48 h, these cells were harvested and lysed. A Dual-Luciferase Reporter Assay system (Promega, USA) was employed to determine the luciferase activity.

### Chromatin immunoprecipitation (ChIP)

ChIP analysis was carried out as previously reported [34]. Briefly, cells were fixed in 1% formaldehyde, and a Bioruptor Sonicator (Diagenode; five cycles of 3 s on/3 s off) was used to fragment the DNA into sizes ranging between 200 and 1000 base pairs. The extracts were immunoprecipitated with protein A/G beads and incubated with antibodies against anti-NF-κB antibody (Cell Signaling Technology; #8242) and normal rabbit IgG (Proteintech Group, Inc, Rosemont, IL, USA; 30000-0-AP). The immunoprecipitated DNA fragment was then purified and validated using PCR analysis.

### Statistical analysis

Data are reported as mean ± standard deviation (SD). Statistical analysis was performed using GraphPad Prism 8.4.2 software (San Diego, CA, USA). Two-tailed unpaired Student’s *t*-test was conducted to compare two groups. ANOVA, combined with Dunnett’s multiple comparisons test, was conducted to compare among multiple groups. The receiver operating characteristic (ROC) curves of each parameter and the combination of serum exosomal DGKK were used to evaluate the performance in differentiating between patients with sepsis-induced lung injury and healthy control subjects. The area under the curve (AUC) was calculated. Kaplan–Meier and Cox’s regression models were used to assess overall survival, and the differences were analyzed by a log-rank test. Statistical significance was considered at *P* < 0.05.

## Results

### Discovery of the differential distribution of DGKK content in serum EVs of patients with sepsis-induced lung injury

**Fig. 1:**
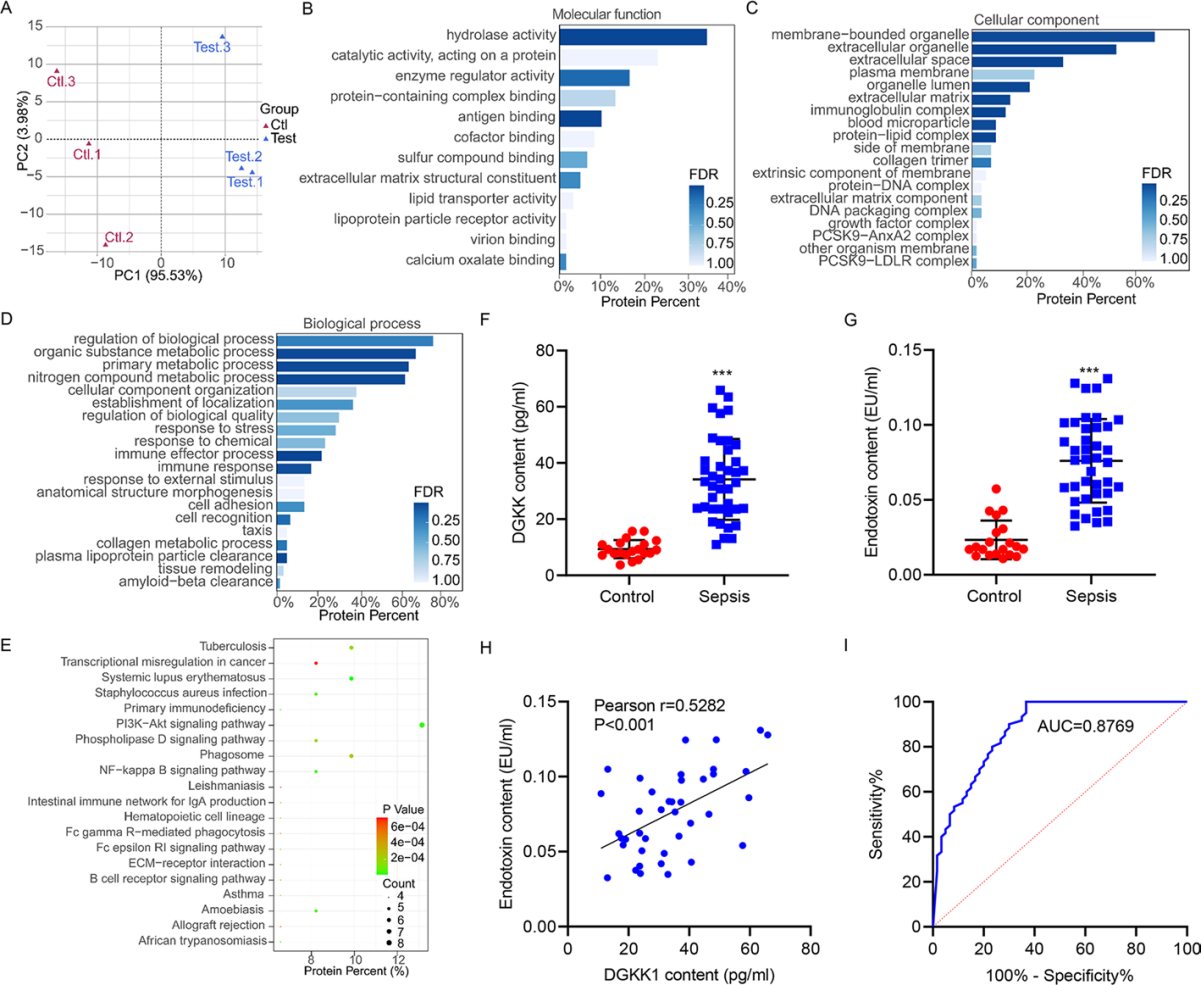
Discovery of DEPs in serum EVs from patients with sepsis-induced lung injury. **A** PLS-DA of protein profiles of EVs isolated from the serum of patients with sepsis-induced lung injury (*n* = 3) and healthy control subjects (*n* = 3). GO analysis of 61 DEPs in the aspects of (**B**) molecular function, (**C**) cellular component, and (**D**) biological process. **E** KEGG enrichment analysis of the 61 DEPs. **F** Isolation of serum EVs and ELISA detection of higher DGKK in patients with sepsis-induced lung injury (*n* = 40) compared with healthy control subjects (*n* = 20). **G** Higher endotoxin content in serum of patients with sepsis-induced lung injury (*n* = 40) compared with healthy control subjects (*n* = 20). **H** Pearson correlation analysis showed positive correlation between serum endotoxin content and exosomal DGKK level in patients with sepsis-induced lung injury (*n* = 40). **I** ROC curve analysis indicating that exosomal DGKK content could be used to differentiate patients with sepsis-induced lung injury from healthy control subjects. ***P < 0.001 versus control

Suspecting altered EVs as a driving factor of oxidative and inflammatory damage in sepsis-induced lung injury, we examined the protein components of EVs from the serum of patients with sepsis-induced lung injury and control subjects. The extraction was confirmed by TEM (Additional file 1: Fig. S1A). Western blot demonstrated that the EVs were positive for CD9, CD81, and TSG101 and negative for Golgi membrane bound protein GM130 (Additional file 1: Fig. S1B). NTA showed the size distribution of EVs (Additional file 1: Fig. S1C). The concentration of serum EVs from healthy controls and septic patients was 3.3 × 10^11^ particles/mL and 3.9 × 10^11^ particles/mL, respectively (Additional file 1: Fig. S1C). LC–MS/MS was performed for serum EV protein profiling. The separation of tests for control subjects and septic patients was confirmed by partial least square discriminant analysis (PLS-DA), where a cumulative variance of 99.5% was shown (component 1 explaining 95.53% and component 2 explaining 3.98% of the variance) (Fig. 1A). We screened the raw data to select those with at least two nonzero values in three repeat experiments for further analysis. Within the 61 proteins identified as differentially distributed between EVs, Gene Ontology (GO) analysis of the molecular function found that hydrolase activity was the most dominant term (Fig. 1B). Similarly, GO analysis of the cellular component found that membrane bound organelle was the most dominant term (Fig. 1C), and GO analysis of the biological process found that several metabolic processes were the major terms (Fig. 1D). KEGG analysis for these 61 proteins revealed several immune-related functions as the mainly enriched terms (Fig. 1E). Among them, proteins showing fold change > 2 (up- or downregulated), and a *t*-test *P*-value < 0.05, were defined as differentially expressed proteins (DEPs) (Additional file 2: Table S1).

Among the 12 proteins that were upregulated in EVs from patients with sepsis-induced lung injury, DGKK was the most significant one. As the DGK family governs a wide range of pathological processes, including oxidative stress and immune response, and DGKK is a critical member of this family, our further approach will be focused on this protein. Elevated level of DGKK in serum EVs from patients with sepsis-induced lung injury was confirmed by ELISA (Fig. 1F). Moreover, the endotoxin content in these patient samples was significantly increased (Fig. 1G). Interestingly, the level of exosomal DGKK and content of serum endotoxin exhibited strong positive correlation shown by Pearson correlation analysis (Fig. 1H). Furthermore, the ROC curves of serum exosomal DGKK were used to differentiate patients with sepsis-induced lung injury from healthy control subjects. In distinguishing patients with sepsis-induced lung injury from healthy control subjects, serum exosomal DGKK demonstrated high diagnostic performance (AUC of 0.8769) (Fig. 1I). The association of exosomal DGKK with a variety of clinicopathological features was also identified (Table 2). Thus, serum exosomal DGKK facilitates tissue damage in the progression of sepsis-induced lung injury.

**Table 2:** Relationship between DGKK expression and clinicopathological features of patients with sepsis-induced lung injury

	Control subjects	Sepsis		*P* value
		DGKK low	DGKK high	
Age Male, *n* (%) BMI (kg/m^2^) White cell count (× 10^9^/L) C-reactive protein (mg/L) IL-6 (pg/mL) TNF-α (pg/mL) SOFA score History of hypertension, *n* (%) History of hyperlipidemia, *n* (%) History of diabetes, *n* (%) History of CKD, *n* (%) History of CCVD, *n* (%) History of asthma, *n* (%) History of COPD, *n* (%)	57.9 ± 8.4 13 (65) 25.5 ± 3.0 5.3 ± 1.0 1.5 ± 0.7 16.0 (10.3–16.3) 40.8 (28.6–49.9) 2 (1–3) 11 (55) 12 (60) 8 (40) 9 (45) 12 (60) 12 (60) 10 (50)	59.9 ± 11.8 8 (40) 25.3 ± 3.1 12.7 ± 0.4 138.5 ± 48.7 41.9 (18.7–55.6) 118.0 (90.2–127.2) 4 (3–5) 8 (40) 8 (40) 13 (65) 13 (65) 7 (35) 8 (40) 6 (30)	65.7 ± 10.3 14 (70) 23.4 ± 2.4 14.5 ± 1.13 169.8 ± 40.6 55.9 (37.3–77.2) 126.4 (103.4–149.0) 7 (5–8) 14 (70) 13 (65) 7 (35) 8 (40) 14 (70) 14 (70) 16 (80)	0.060 0.119 0.054 < 0.001 < 0.001 < 0.001 < 0.001 < 0.001 0.162 0.243 0.125 0.247 0.072 0.150 0.006

Results expressed as mean ± SD or median (interquartile range)

*CKD* chronic kidney disease, *CCVD* cardiovascular and cerebrovascular diseases, *COPD* chronic obstructive pulmonary disease, *SOFA* sequential organ failure assessment

### DGKK was expressed in CD4^+^ T cells through the activation of the NF-κB pathway

Examining the expression of DGKK in various tissues and cell types from a public database (http://www.proteinatlas.org/), we found that DGKK is mainly expressed in T lymphocytes. The isolated CD4^+^ T cells from healthy control subjects exhibited elevated expression of DGKK in cell lysates under LPS challenge at those mRNA (Additional file 1: Fig. S2A) and protein (Additional file 1: Fig. S2B) levels. Treatment with NF-κB inhibitor QNZ strongly suppressed the activation of the NF-κB pathway in CD4^+^ T cells, as indicated by the abundance of TLR4 and cytoplasmic/nuclear distribution of NF-κB p65 (Additional file 1: Fig. S2C). With this treatment, LPS no longer enabled DGKK expression (Additional file 1: Fig. S2D). This was further supported by luciferase reporter assay to determine the activation of promoter elements of DGKK gene, which showed that LPS treatment dramatically induced reporter expression, but QNZ treatment, or use of a mutant promoter element, restrained this increase (Additional file 1: Fig. S2E). The NF-κB binding site in the DGKK promoter was predicted using JASPAR [35] (Additional file 1: Fig. S2F). ChIP–qPCR analysis based on the predicted binding site showed that p65 binding was enhanced by LPS treatment and that QNZ limited this increase (Additional file 1: Fig. S2G).

### EVs derived from the serum of patients with sepsis-induced lung injury and LPS-treated CD4^+^ T cells promoted sepsis-induced lung injury in mice

**Fig. 2:**
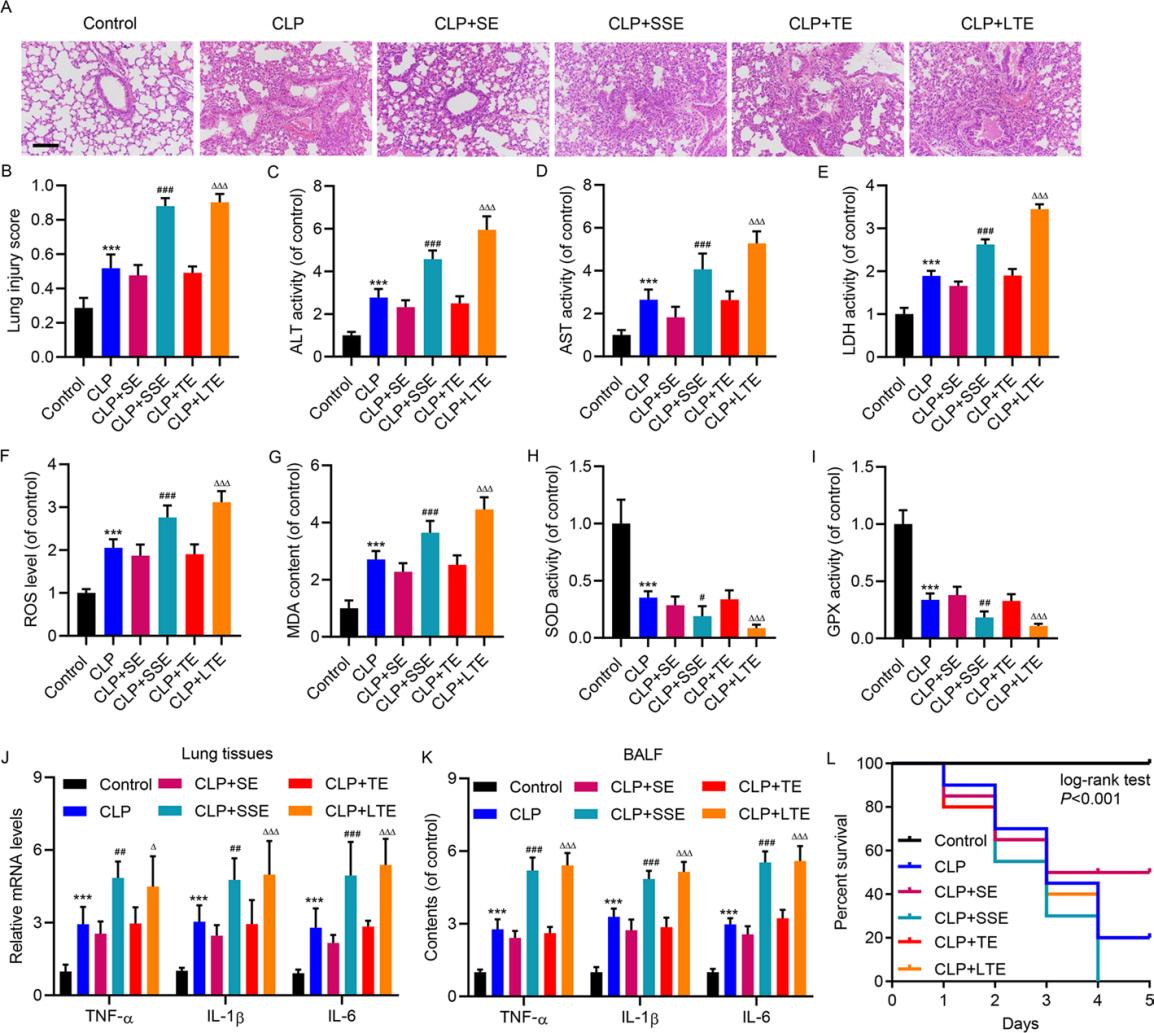
Toxic effects of CD4^+^ T cell-derived EVs on oxidative stress and inflammation in CLP-induced lung injury in mice. CLP model mice were treated with EVs isolated from the serum of patients with sepsis-induced lung injury (SSE) or of healthy subjects (SE), or CD4^+^ T cells isolated from healthy subjects treated with (LTE) or without (TE) 10 μg/mL LPS. **A** H and E staining (scale bar, 100 μm). **B** Severity of histological injury. Plasma levels of (**C**) ALT, (**D**) AST, (**E**) LDH, and (**F**) ROS level, **G** MDA content, **H** SOD activity, (**I**) GPX activity and (**J**) mRNA expression of TNF-α, IL-1β, and IL-6 in lung tissues were measured. **K** The BALF content of TNF-α, IL-1β, and IL-6 in mice was measured by ELISA. **L** The survival rate of mice was monitored within 5 days, showing shortened survival with EV treatment. Data presented as mean ± SD. ***P < 0.001 versus control. ^#^*P* < 0.05, ^##^*P* < 0.01, ^###^*P* < 0.001 versus CLP + SE. ^Δ^*P* < 0.05, ^ΔΔΔ^*P* < 0.001 versus CLP + TE

How CD4^+^ T cells affect immune response and the whole process of pathogenesis in sepsis-induced lung injury remains to be elucidated. The present study is focused on exosomal function, so EVs were extracted from the serum of patients with sepsis-induced lung injury (and control subjects) or cultured primary human CD4^+^ T cells treated with LPS. The quality of EVs extracted from CD4^+^ T cells, with or without LPS treatment, was examined by TEM (Additional file 1: Fig. S3A) and western blot of exosomal markers (Additional file 1: Fig. S3B). NTA showed the size distribution of EVs (Additional file 1: Fig. S3C). The concentration of EVs derived from CD4^+^ T cells with or without LPS treatment was 5.3 × 10^9^ particles/mL and 1.6 × 10^9^ particles/mL, respectively (Additional file 1: Fig. S3C). Sepsis was induced by CLP in mice, where the pathological examination by H and E staining showed substantial morphological changes, including edema, hemorrhage, alveolar collapse, and inflammatory cell infiltrations, compared with the control group (Fig. 2A, B). With injection of the above-mentioned EVs, the progression of lung injury was enhanced by EVs from patients with sepsis-induced lung injury and from LPS-treated CD4^+^ T cells compared with EVs from control subjects and from untreated CD4^+^ T cells (Fig. 2A, B). Plasma levels of ALT, AST, and LDH were significantly elevated in the CLP group compared with the control group (Fig. 2C–E). Moreover, EVs from patients with sepsis-induced lung injury and LPS-treated CD4^+^ T cells further increased the levels of ALT, AST, and LDH in CLP mice compared with EVs from control subjects and from untreated CD4^+^ T cells (Fig. 2C–E). ROS measurement of lung tissue showed that EVs from patients with sepsis-induced lung injury and from LPS-treated CD4^+^ T cells further elevated ROS levels in CLP mice compared with EVs from control subjects and from untreated CD4^+^ T cells (Fig. 2F). A similar trend was also observed when the MDA content, a marker of ROS damage, was monitored (Fig. 2G). Consistently, the antioxidant activity, indicated by SOD (Fig. 2H) and GPX activities (Fig. 2I), showed further reduction in CLP model mice treated with EVs from patients with sepsis-induced lung injury and from LPS-treated CD4^+^ T cells compared with EVs from control subjects and from untreated CD4^+^ T cells. Similar to oxidative stress markers, the inflammatory cytokines (TNF-α, IL-1β, and IL-6) showed further elevation in CLP model mice treated with EVs from patients and LPS-treated T cells compared with EVs from control subjects and control T cells. RT-qPCR and ELISA were used to measure inflammatory cytokines in lung tissues (Fig. 2J) and BALF (Fig. 2K), respectively. Consistent with these observations, treatment with EVs from patients and LPS-treated T cells, compared with EVs from control subjects and control T cells, further shortened the life of CLP model mice (Fig. 2L). Moreover, the lung injury, oxidative stress, and inflammation induced by EVs isolated from the above-mentioned serum and CD4^+^ T cells were comparable with those in CLP-treated mice (Additional file 1: Fig. S4A–G). Taken together, these data suggest that EVs derived from the serum of patients with sepsis-induced lung injury and LPS-treated CD4^+^ T cells promoted sepsis-induced lung injury.

### CD4^+^ T cells secreted EVs to induce A549 cell apoptosis and oxidative stress in vitro

**Fig. 3:**
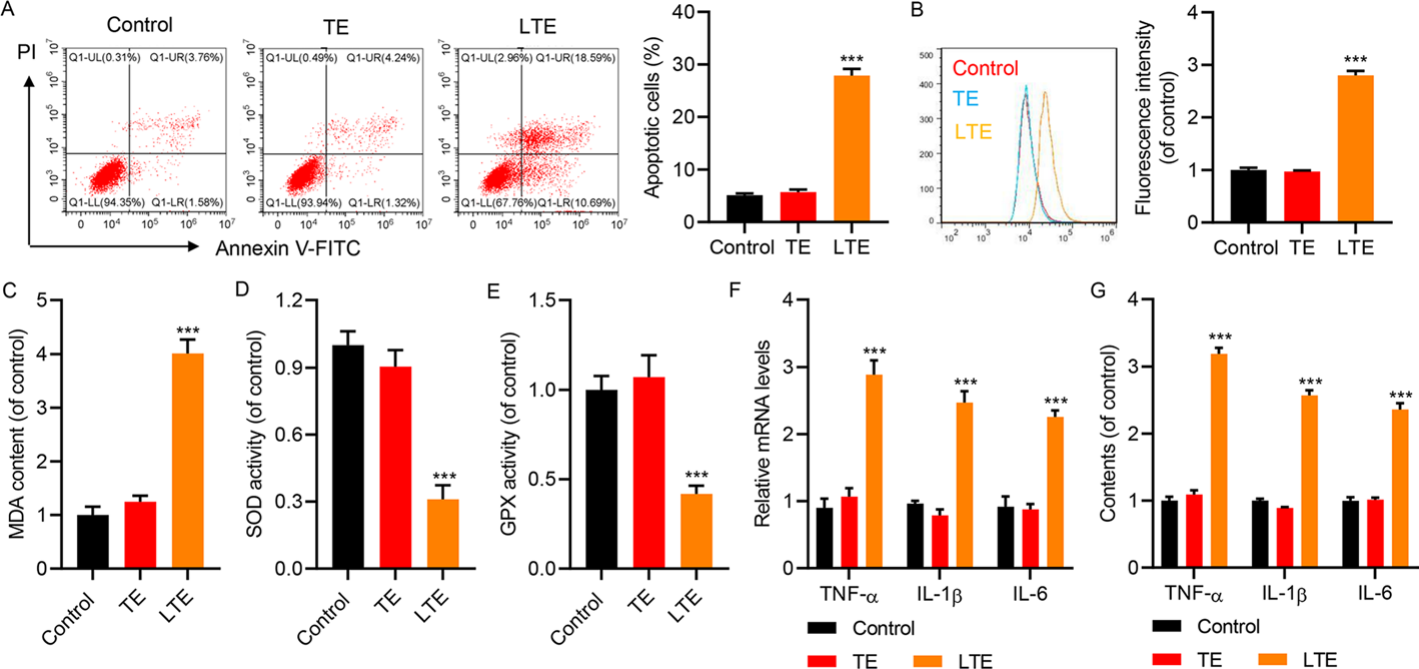
Toxic effects of CD4^+^ T-cell-derived EVs on oxidative stress and inflammation on apoptosis, oxidative stress, and inflammation in A549 cells. A549 cells were treated with EVs isolated from CD4^+^ T cells isolated from healthy subjects treated with (LTE) or without (TE) 10 μg/mL LPS. **A** Flow cytometric analysis with Annexin V-FITC/PI double staining indicated elevation of cell apoptosis by LTE treatment. Representative plot images from flow cytometry and statistical analysis are shown. **B** ROS production measured by DCFH-DA staining with flow cytometric analysis showed higher ROS in cells treated with LTE. Representative images from flow cytometry and statistical analysis are shown. **C** Higher MDA content in cells treated with LTE. **D** SOD and (**E**) GPX activities were reduced in cells treated with LTE. Elevated (**F**) mRNA expression and (**G**) secretion of TNF-α, IL-1β, and IL-6 in cells treated with LTE. Data presented as mean ± SD. ***P < 0.001 versus control

In light of the findings in CLP model mice, we further examined the effects of CD4^+^ T cell-secreted EVs on cultured lung epithelial cells. A549 cells, the cell line of adenocarcinomic human alveolar basal epithelial cells, were cultured and treated with EVs derived from CD4^+^ T cells with or without LPS. Laser scanning confocal microscope analysis of EV uptake is shown in Additional file 1: Fig. S3D. Flow cytometric analysis with Annexin V-FITC/PI double staining indicated that EVs from LPS-treated T cells, but not from control T cells, dramatically promoted cell apoptosis (Fig. 3A). The ROS levels in these cells detected by DCFH-DA staining with flow cytometric analysis revealed that EVs from LPS-treated T cells, but not from control T cells, dramatically exaggerated ROS production (Fig. 3B). MDA content was also elevated in A549 cells treated with EVs from LPS-treated T cells, but not from control T cells (Fig. 3C). The activities of SOD (Fig. 3D) and GPX (Fig. 3E) were reduced in A549 cells treated with EVs from LPS-treated T cells, but not from control T cells. Measured by RT-qPCR analysis for total mRNA (Fig. 3F) and ELISA for cell culture medium (Fig. 3G), the expression and secretion of inflammatory cytokines (TNF-α, IL-1β, and IL-6) were elevated in A549 cells treated with EVs from LPS-treated T cells, but not from control T cells. Therefore, LPS-treated CD4^+^ T-cell-secreted EVs promoted the apoptosis of alveolar epithelial cells, accompanied with the elevation of ROS and inflammatory reaction, which could be critical factors to drive lung injury.

### PKC/NOX4 pathway mediated the toxic effects of CD4^+^ T-cell-derived EVs on cultured alveolar epithelial cells

Encouraged by the findings of elevated distribution of CD4^+^ T-cell-expressed DGKK in serum EVs from patients with sepsis-induced lung injury, the downstream pathway of DGK was examined for the involvement in the toxic effects of LPS-treated CD4^+^ T-cell-derived EVs on cultured A549 cells. DGK phosphorylates DAG to generate PA, which could activate the PKC pathway. Consistently, the stimulation of EVs isolated from LPS-treated and control CD4^+^ T cells to A549 cells strongly enhanced DAG content (Additional file 1: Fig. S5A), accompanied with the activation of PKC (Additional file 1: Fig. S5B). As PKC phosphorylates p40phox to activate NADPH oxidase (NOX), the expression of NOX enzymes (NOX1-5 and DUOX1/2) was examined. The results showed that only NOX4 exhibited induction by EVs from LPS-treated CD4^+^ T cells (Additional file 1: Fig. S5C). The upregulation of NOX4 was also detected by western blot (Additional file 1: Fig. S5D), suggesting that NOX4 may be involved in the toxic effects of EVs derived from CD4^+^ T cells treated with LPS.

**Fig. 4:**
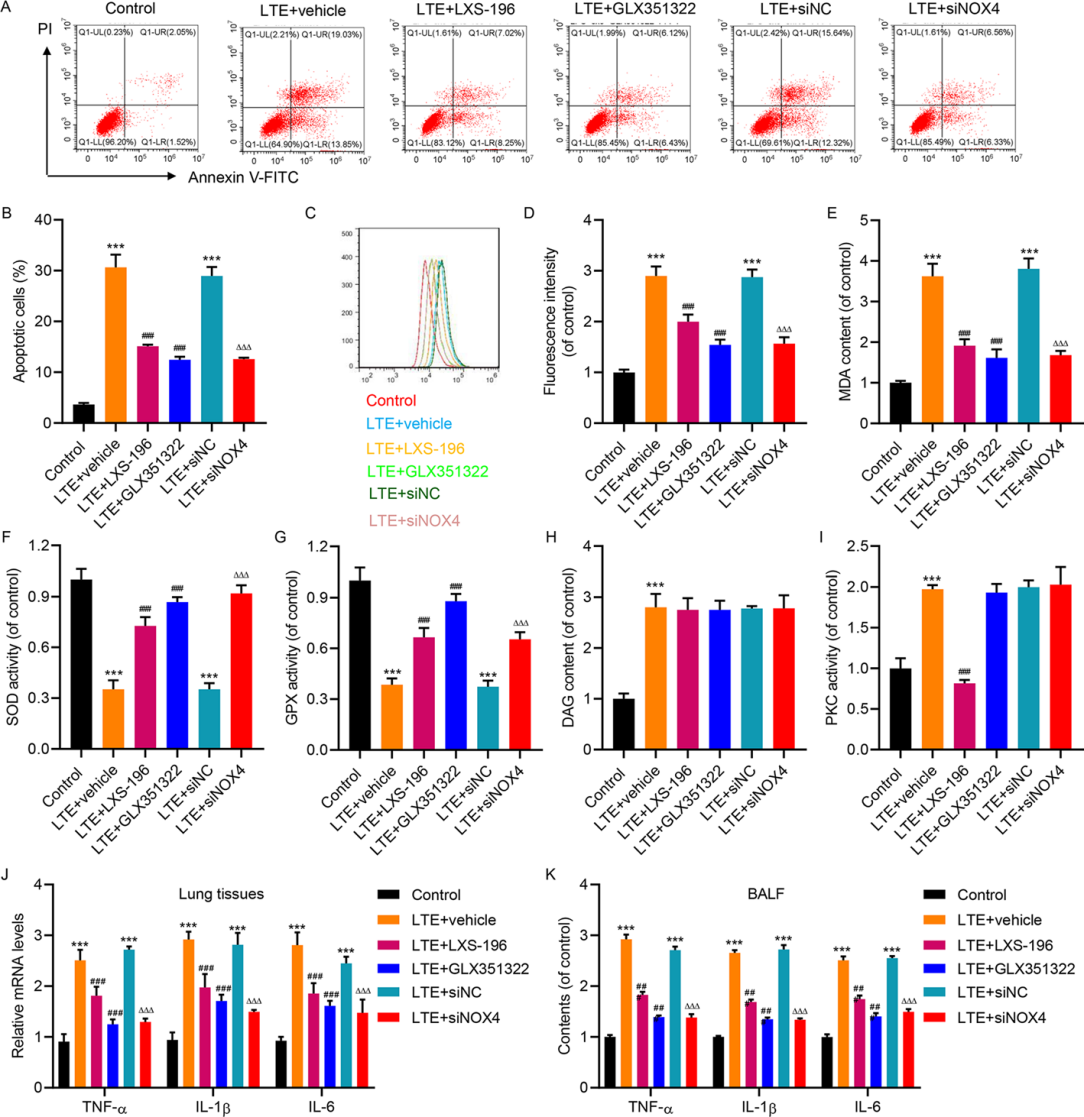
Effects of T cell EVs on apoptosis, oxidative stress, and inflammation in A549 cells were attenuated by PKC/NOX4 inhibition. A549 cells were stimulated with EVs isolated from CD4^+^ T cells isolated from healthy subjects treated with 10 μg/mL LPS (LTE) in the absence or presence of LXS-196, GLX351322, or NOX4 siRNA (taking siRNA to nonspecific sequence as control). **A**, **B** Flow cytometric analysis with Annexin V-FITC/PI double staining indicated that the elevation of cell apoptosis by LTE treatment was restored by LXS-196, GLX351322, or NOX4 RNAi. Representative plot images from flow cytometry (**A**) and statistical analysis (**B**) are shown. **C**, **D** DCFH-DA staining with flow cytometric analysis indicated that the elevation of ROS by LTE treatment was restored by LXS-196, GLX351322, or NOX4 RNAi. Representative images from flow cytometry (**C**) and statistical analysis (**D**) are shown. **E** The elevation of MDA content by LTE treatment was restored by LXS-196, GLX351322, or NOX4 RNAi. The reduction of SOD (**F**) and GPX activities (**G**) by LTE treatment was restored by LXS-196, GLX351322, or NOX4 RNAi. **H** The elevation of DAG content by LTE treatment was not affected by LXS-196, GLX351322, or NOX4 RNAi. **I** The elevation of PKC activity by LTE treatment was restored by LXS-196, but was not affected by GLX351322 or NOX4 RNAi. The increase of (**J**) mRNA expression and (**K**) secretion of TNF-α, IL-1β, and IL-6 by LTE treatment was restored by LXS-196, GLX351322, or NOX4 RNAi. Data presented as mean ± SD. ***P < 0.001 versus control. ^###^*P* < 0.001 versus LTE + vehicle. ^ΔΔΔ^*P* < 0.001 versus LTE + siNC

To determine the role of the PKC pathway in the toxic effects of LPS-treated CD4^+^ T-cell-derived EVs, the A549 cells were treated with the selective PKC inhibitor LXS-196 (Darovasertib) or a novel NOX4 inhibitor GLX351322, together with the EVs. Flow cytometric analysis with Annexin V-FITC/PI double staining indicated the dramatic induction of apoptosis by those EVs, but LXS-196 and GLX351322 both attenuated the apoptosis induction (Fig. 4A, B). NOX4 could be specifically depleted through siRNA transfection (Additional file 1: Fig. S5E, F), and this knockdown of NOX4 also weakened the toxic effects of LPS-treated CD4^+^ T-cell-derived EVs (Fig. 4A, B). In parallel, the elevated ROS levels (Fig. 4C, D) and MDA content (Fig. 4E) in EV-treated A549 cells were also reduced by LXS-196, GLX351322, and siRNA of NOX4. The reduction of SOD (Fig. 4F) and PGX activities (Fig. 4G) in EV-treated A549 cells was restored by LXS-196, GLX351322, and NOX4 RNAi. As LXS-196, GLX351322, and NOX4 RNAi all worked downstream of DAG, the elevation of DAG content in EV-treated A549 cells was not affected by LXS-196, GLX351322, or NOX4 RNAi (Fig. 4H). LXS-196 efficiently suppressed the elevation of PKC activity in EV-treated A549 cells (Fig. 4I). The expression (Fig. 4J) and secretion (Fig. 4K) of inflammatory cytokines (TNF-α, IL-1β, and IL-6) were examined. The results showed that LXS-196, GLX351322, and NOX4 RNAi exerted inhibitory effects on the upregulation of these cytokines in EV-treated A549 cells. Together, the PKC/NOX4 pathway acts downstream of CD4^+^ T-cell-derived EVs in cultured alveolar epithelial cells to induce oxidative stress, inflammatory response, and cell apoptosis.

### PKC/NOX4 pathway mediated the toxic effects of CD4^+^ T-cell-derived EVs in mice with sepsis-induced lung injury

**Fig. 5:**
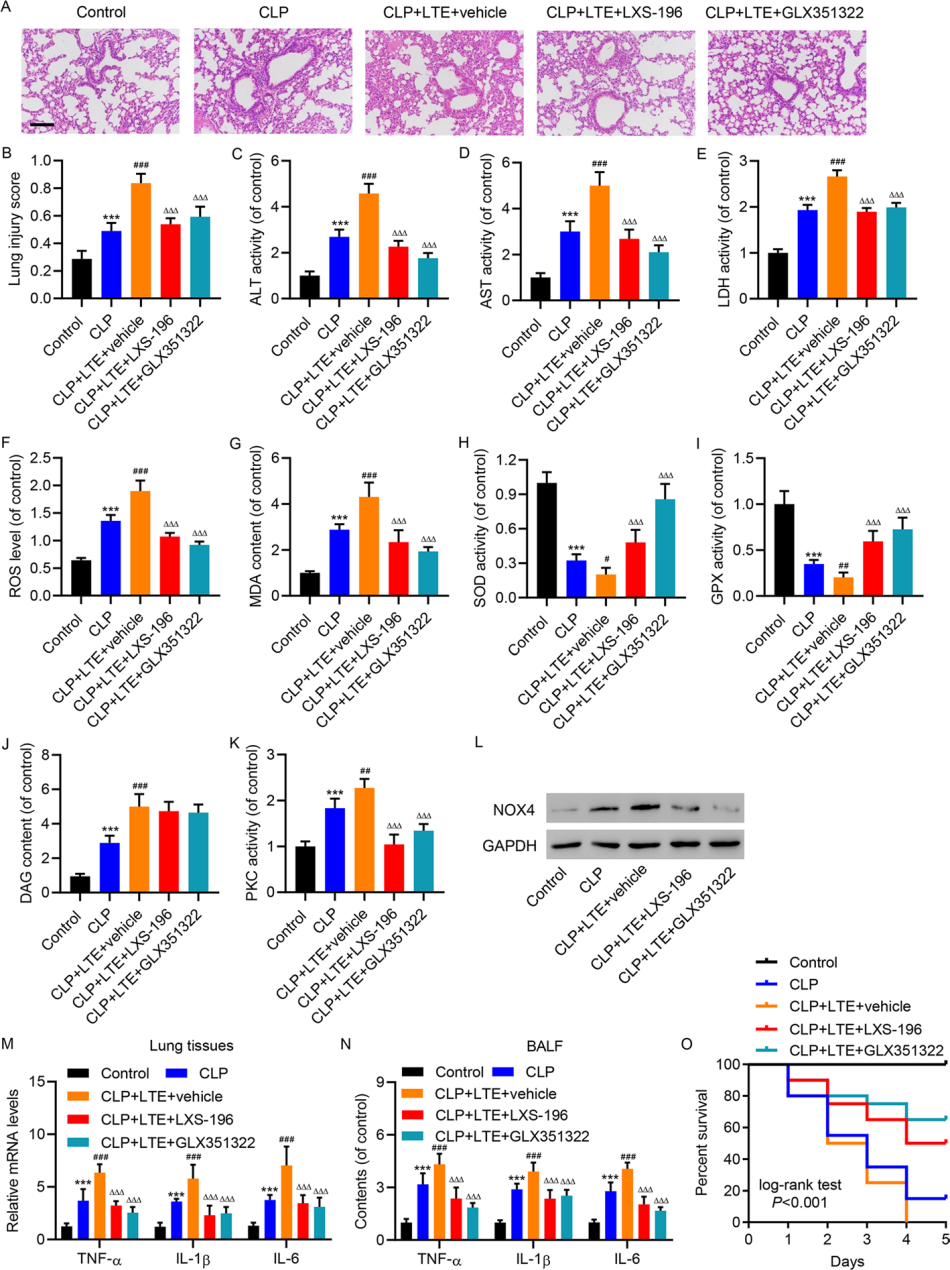
Effects of T-cell EVs on tissue damage, oxidative stress, and inflammation in mice with CLP-induced lung injury were attenuated by PKC/NOX4 inhibition. Mice with CLP were stimulated with EVs isolated from CD4^+^ T cells isolated from healthy subjects treated with 10 μg/mL LPS (LTE), in the absence or presence of LXS-196 or GLX351322. **A** H and E staining indicated that the severe lung injury by LTE treatment in CLP model mice was restored by LXS-196 or GLX351322 (scale bar, 100 μm). **B** The severity of histological injury and plasma levels of (**C**) ALT, (**D**) AST, and (**E**) LDH by LTE treatment in CLP model mice was restored by LXS-196 or GLX351322. The elevation of (**F**) ROS levels and (**G**) MDA content by LTE treatment in CLP model mice was restored by LXS-196 or GLX351322. The reduction of (**H**) SOD and (**I**) GPX activities by LTE treatment in CLP model mice was restored by LXS-196 and GLX351322. **J** The elevation of DAG content by LTE treatment in CLP model mice was not affected by LXS-196 or GLX351322. **K** The elevation of PKC activity by LTE treatment in CLP model mice was restored by LXS-196, but not GLX351322. **L** The elevation of NOX4 protein level by LTE treatment in CLP model mice was restored by LXS-196 and GLX351322, as shown by western blot. **M** The elevation of mRNA expression of TNF-α, IL-1β, and IL-6 by LTE treatment in CLP model mice was restored by LXS-196 or GLX351322. **N** The elevation of BALF content of TNF-α, IL-1β, and IL-6 by LTE treatment in CLP model mice was restored by LXS-196 or GLX351322. **O** The shortened survival in CLP model mice treated with LTE was rescued by LXS-196 and GLX351322. Data presented as mean ± SD. ***P < 0.001 versus control. ^#^*P* < 0.05, ^##^*P* < 0.01, ^###^*P* < 0.001 versus CLP. ^ΔΔΔ^*P* < 0.001 versus CLP + LTE + vehicle

To further elucidate the action of the PKC/NOX4 pathway downstream of LPS-treated CD4^+^ T-cell-derived EVs in sepsis-induced lung injury, we directed our attention to CLP-induced sepsis in mice. The toxic effects of EVs were confirmed by H and E staining of lung tissue in CLP model mice, and the protective effects of LXS-196 and GLX351322 were observed (Fig. 5A, B. The increasing plasma levels of ALT, AST, and LDH by treatment of EVs in CLP model mice were weakened by LXS-196 and GLX351322 (Fig. 5C–E). The increasing ROS levels (Fig. 5F) and MDA content (Fig. 5G) by treatment of EVs in CLP model mice were weakened by LXS-196 and GLX351322. Correspondingly, the activities of SOD (Fig. 5H) and GPX (Fig. 5I) were further reduced by treatment of EVs in CLP model mice, which were restored by LXS-196 and GLX351322. Neither LXS-196 nor GLX351322 had an effect on DAG content in CLP model mice with treatment of EVs (Fig. 5J). Only LXS-196 exerted inhibitory effect on PKC activity in CLP model mice with treatment of EVs (Fig. 5K). The induction of NOX4 expression in the lungs by CLP and treatment of EVs, and the inhibitory effects of LXS-196 and GLX351322 were demonstrated by western blot (Fig. 5L). After examining the mRNA expression (Fig. 5M) and BALF content (Fig. 5N) of inflammatory cytokines (TNF-α, IL-1β, and IL-6), LXS-196 and GLX351322 showed inhibitory effects on the upregulation of these cytokines in EV-treated CLP model mice. Consistently, the shortened survival in CLP model mice, and CLP model mice treated with EVs, was rescued by LXS-196 and GLX351322 (Fig. 5O).

**Fig. 6:**
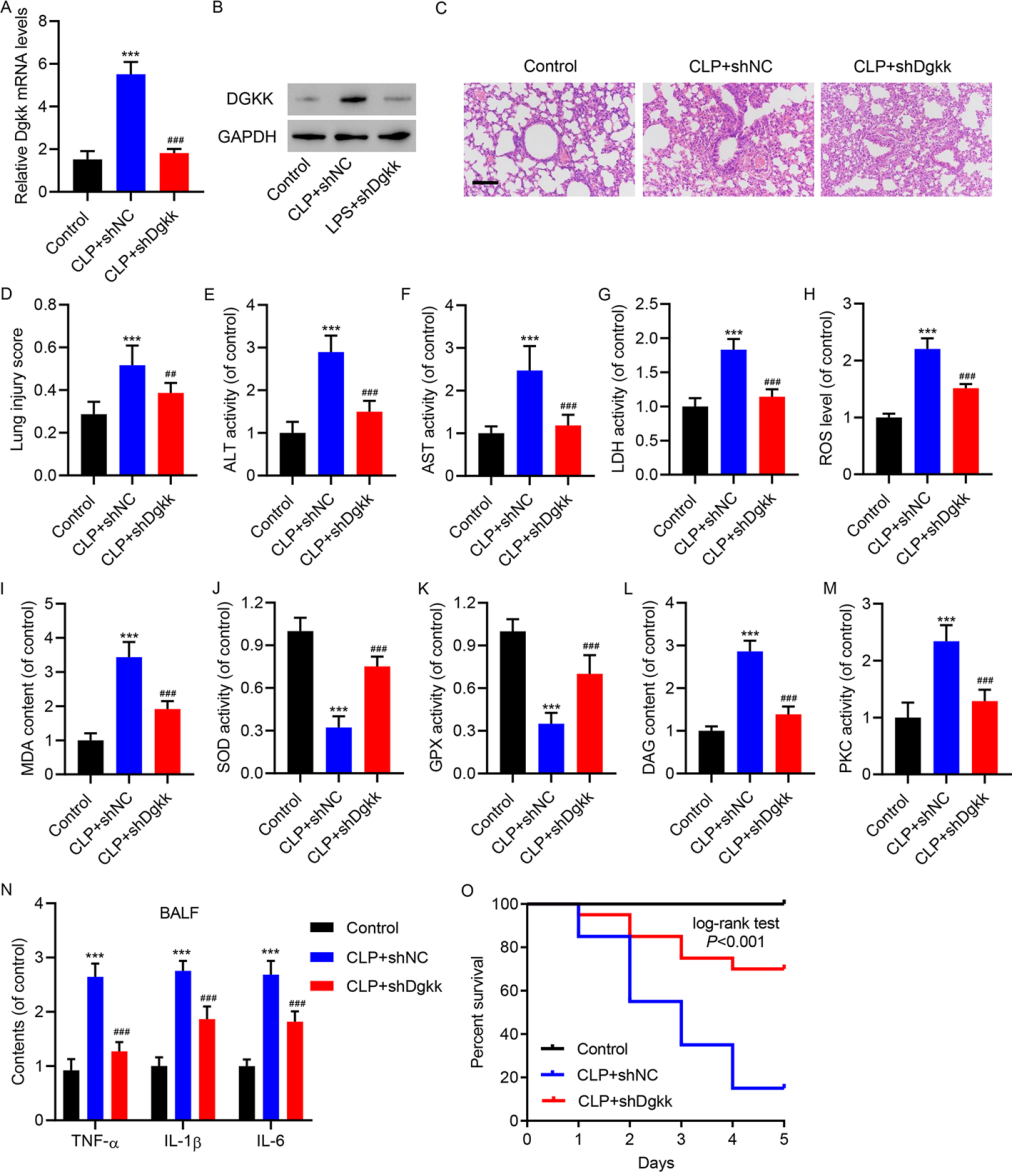
DGKK knockdown attenuated tissue damage, oxidative stress, and inflammation in mice with CLP-induced lung injury. Mice with CLP were injected with virus carrying plasmid expressing shRNA control (shNC) and shRNA targeting DGKK (shDgkk). The elevation of Dgkk abundance at the (**A**) mRNA and (**B**) protein levels in CLP model mice was restored by Dgkk RNAi. **C** H and E staining indicated that the severe lung injury in CLP model mice was restored by Dgkk RNAi (scale bar, 100 μm). **D** The severity of histological injury and plasma levels of (**E**) ALT, (**F**) AST, and (**G**) LDH in CLP model mice was restored by Dgkk RNAi. The elevation of (**H**) ROS levels and (**I**) MDA content in CLP model mice was restored by Dgkk RNAi. The reduction of (**J**) SOD and (**K**) GPX activities in CLP model mice was restored by Dgkk RNAi. The elevation of (**L**) DAG content and (**M**) PKC activity in CLP model mice was restored by Dgkk RNAi. **N** The elevation of BALF content of TNF-α, IL-1β, and IL-6 in CLP model mice was restored by Dgkk RNAi. **O** The shortened survival in CLP model mice was restored by Dgkk RNAi. Data presented as mean ± SD. ***P < 0.001 versus control. ^##^*P* < 0.01, ^###^*P* < 0.001 versus CLP + shNC

The toxic effects of EVs, confirmed by H and E staining for lung tissue in CLP model mice, were also ameliorated by injection of virus expressing shRNA control and shRNA targeting DGKK (Fig. 6A–D). The increasing plasma levels of ALT, AST, and LDH in CLP model mice were decreased by DGKK RNAi (Fig. 6E–G). The increase of ROS levels (Fig. 6H) and MDA content (Fig. 6I) in CLP model mice were decreased by DGKK RNAi. Correspondingly, the reduced activity of SOD (Fig. 6J) and GPX (Fig. 6K) was restored by DGKK RNAi. As expected, DGKK RNAi efficiently inhibited the elevation of DAG content (Fig. 6L) and PKC activity (Fig. 6M) in the lungs of CLP model mice. The increased BALF content of inflammatory cytokines (TNF-α, IL-1β, and IL-6) in CLP model mice was also decreased by DGKK RNAi (Fig. 6N). The shortened survival in CLP model mice was restored by DGKK RNAi (Fig. 6O). Therefore, CD4^+^ T-cell-derived EVs might place unique demands on the DGK/DAG/PKC/NOX4 pathway to promote sepsis-induced lung injury in mice, including oxidative stress and inflammation.

## Discussion

In lung tissue affected by ALI, many sources generate ROS, including itinerant and resident leukocytes, parenchymal cells, oxidant-generating enzymes in the blood, and inhaled high-oxygen gases from mechanical ventilation. ROS facilitate tissue damage in ALI with prolonged inflammatory response [4]. However, the upstream regulators of inflammation and oxidative stress supporting the pathogenesis of sepsis-induced ALI are not systematically studied. The present approach expanded this knowledge by elucidating the DGKK/PKC/NOX4 pathway. As DGKK was delivered to target tissue by T-cell-derived EVs in patients with ALI, this study further underscored the importance of EVs in orchestrating pathogenesis of ALI/ARDS.

In sepsis-induced lung injury, EVs from various cell types exert broad protective or promoting effects on tissue damage. For example, EVs derived from human mesenchymal stem cells effectively downregulated sepsis-induced glycolysis and inflammation in macrophages, which could attenuate lung damage and improve the survival of septic mice [36]. In particular, adipose-derived MSC-derived EVs could inhibit IL-27 secreted from macrophages, which ameliorates sepsis-induced ALI in model mice [37]. In addition, exosomal miR-30d-5p from polymorphonuclear neutrophils is involved in sepsis-induced ALI by inducing M1 polarization and pyroptosis of macrophages [38]. However, no studies have reported on the functional involvement of EVs secreted from CD4^+^ T cells.

The EVs produced by activated CD4^+^ T cells not only express similar proteins as in other kinds of EVs, including membrane-anchored tetraspanins, annexins, and representative luminal proteins, but also express immune-related proteins, including integrins, HLA-I, microglobulin, and TCR/CD3 complex subunits. EVs have been associated with various chronic inflammatory lung diseases. For example, EVs derived from lung tissue carry miR-210 that prevents the expression of Atg7 in target cells to prevent autophagy and stimulate myofibroblast differentiation and fibrosis in COPD [7]. CD36^+^ EVs accelerate disease progression by activating inflammation through the heterodimerization of TLR4/6 in asthma [39]. EVs from BALF of patients with asthma contain functional leukotriene-producing enzymes that cause the secretion of inflammatory cytokines by bronchial epithelial cells [8]. EVs derived from infiltrated and activated neutrophils and eosinophils are also proinflammatory [9]. On the basis of this premise, we hypothesized that T-cell-derived EVs could carry important factors to support sepsis-induced lung injury, particularly to promote inflammation and oxidative stress. Through proteomic profiling, 61 DEPs were found in serum EVs from patients with sepsis-induced lung injury compared with serum EVs from healthy control subjects. Among them, DGKK was further examined.

DGK family kinases, which comprise diverse isozymes (α–κ), take part in the pathogenesis of normal and abnormal biological processes, including immune responses, neuronal network activation, brain disorder, cancer, and type 2 diabetes [18]. DGK phosphorylates DAG to activate PKC pathways, followed by NOX activation. The inhibition of PKC or NOX4 attenuated *Pseudomonas aeruginosa*-induced lung inflammatory injury by inhibiting ROS production [40] and LPS-induced ALI by inhibiting apoptosis and secretion of proinflammatory cytokines in pneumonia cells [41]. Serum apelin-13 could protect against sepsis-induced ALI by regulating NOX4-dependent ROS [42]. NOX4 (versus other NOX isoforms) was specifically involved in damage of the endothelial cell barrier in the lungs of a mouse model of CLP-induced sepsis [43]. Echoing these previous reports, our investigation demonstrated that NOX4 was specifically regulated by the DAG/PKC axis, and this DAG/PKC/NOX4 signaling played a supportive role in sepsis-induced lung injury, again indicating the therapeutic value of this signaling in the treatment of this disease.

Overall, the present study systematically analyzed the protein profile of serum EVs in patients with sepsis-induced lung injury. DGKK was expressed in CD4^+^ T cells under regulation of the NF-κB pathway and carried by EVs for delivery to target cells. In the cultured alveolar epithelial A549 cell line and the lungs of a mouse model of CLP-induced sepsis, EVs derived from CD4^+^ T cells exerted toxic effects through DGKK and its stimulation on the DAG/PKC/NOX4 signaling pathways. As there are still other proteins identified with altered distribution in serum EVs of patients with sepsis-induced lung injury, future studies need to elucidate the involvement of these other proteins in the pathogenesis. Moreover, DGK maintains the balance between DAG and PA. The current study was focused on the signaling pathways downstream of DAG, but the PA-initiated signal might also be involved in lung injury in sepsis, which needs to be further studied. Nevertheless, the findings reported here could still be leveraged to develop treatment strategies for patients with ALI/ARDS.

## Conclusions

Our findings demonstrate that the upregulated levels of DGKK in serum EVs derived from septic patients showed strong correlation with sepsis severity and progression. Mechanistically, EVs derived from LPS-treated CD4^+^ T cells carrying DGKK induced oxidative stress and inflammation in alveolar epithelial A549 cells and sepsis-induced mice through PKC and NOX4, the downstream effectors of DGKK and DAG (Additional file 1: Fig. S6). This approach established the mechanism that T-cell-derived EVs exerted toxic effects in sepsis-induced lung injury through the DGKK/DAG/PKC/NOX4 pathway.

## Supplementary Information

**Additional file 1: Figure S1.** Characterization of EVs isolated from the serum of patients with sepsis-induced lung injury and healthy control subjects. **A** TEM observation (scale bar, 200 nm). **B** Western blot analysis of EV markers. **C** Diameter distribution of EVs by NTA. **Figure S2.** LPS promoted DGKK expression via the NF-κB pathway in human CD4^+^ T cells. Elevation of DGKK expression at the **A** mRNA and **B** protein levels in CD4^+^ T cells isolated from healthy subjects treated with 10 μg/mL LPS at various treatment durations. **C** Treatment of QNZ strongly suppressed the upregulation of TLR4 and higher nuclear/cytoplasmic distribution of NF-κB p65 in LPS-treated CD4^+^ T cells, as shown by western blot. **D** The elevation of DGKK expression at the mRNA and protein levels in human CD4^+^ T cells treated with LPS was restored by QNZ. **E** Luciferase reporter assay showed that the WT promoter of the DGKK gene was activated by LPS treatment, which could be restored by QNZ. However, the mutant promoter of the DGKK gene could not be activated by LPS. **F** The NF-κB binding site in the DGKK promoter was predicted using JASPAR. **G** ChIP–qPCR showed that the elevation of NF-κB binding to the DGKK promoter by LPS treatment was restored by QNZ. ***P < 0.001 versus 0 h or control. ^###^*P* < 0.001 versus LPS **Figure S3.** Characterization of EVs isolated from cultured human CD4^+^ T cells treated with or without 10 μg/mL LPS. **A** TEM observation (scale bar, 200 nm). **B** Western blot analysis of EV markers and DGKK. **C** Diameter distribution of EVs by NTA. **D** Laser scanning confocal microscope analysis of EV uptake by A549 cells (scale bar, 50 μm). **Figure S4**. Toxic effects of CD4^+^ T-cell-derived EVs on oxidative stress and inflammation in mice. Mice were treated with CLP, SSE, SE, LTE, or TE. **A** H and E staining (scale bar, 100 μm). **B** Severity of histological injury. Plasma levels of **C** ALT, **D** AST, **E** LDH, and **F** ROS level, **G** MDA content, **H** SOD activity, **I** GPX activity, and **I** BALF content of TNF-α, IL-1β, and IL-6 in lung tissues of mice. **K** The survival rate of mice was monitored within 5 days, showing the shortened survival with EV treatment. Data presented as mean ± SD. ***P < 0.001 versus control. ^##^*P* < 0.01, ^###^*P* < 0.001 versus SE. ^Δ^*P* < 0.05, ^ΔΔ^*P* < 0.01, ^ΔΔΔ^*P* < 0.001 versus TE **Figure S5.** Activation of the DAG/PKC/NOX4 signaling pathway by T-cell EVs. A549 cells were treated with EVs isolated from CD4^+^ T cells isolated from healthy subjects treated with (LTE) or without (TE) 10 μg/mL LPS. **A** DAG content and **B** PKC activity were increased by LTE. **C** The mRNA expression of NOX4 in A549 cells was upregulated by LTE, but not NOX1, NOX2, NOX3, NOX5, DUOX1, and DUOX2. **D** The protein level of NOX4 in A549 cells was upregulated by LTE. The expression of NOX4 in A549 cells was significantly suppressed by NOX4 siRNA at the **E** mRNA and **F** protein levels. Data presented as mean ± SD. ***P < 0.001 versus control or siNC **Figure S6.** Schematic representation of the regulation of oxidative stress and inflammation in lung injury by EVs from CD4^+^ T cells via the DGKK/DAG/PKC/NOX4 pathway

**Additional file 2: Table S1.** Differently expressed proteins between patients with sepsis lung injury and healthy subjects by using LC–MS/MS analysis

## Data Availability

The datasets used and/or analyzed during the current study are available from the corresponding author.

## Abbreviations

ALI: Acute lung injury
ARDS: Acute respiratory distress syndrome
LC–MS/MS: Liquid chromatography–tandem mass spectrometry
BALF: Bronchoalveolar lavage fluids
ROS: Reactive oxygen species
SOD: Superoxide dismutase
COPD: Chronic obstructive pulmonary disease
DGK: Diacylglycerol kinase
DAG: Diacylglycerol
PA: Phosphatidic acid
PKC: Rho/protein kinase C
DGKK: DGK kappa
LPS: Lipopolysaccharide
FBS: Fetal bovine serum
PBS: Phosphate-buffered saline
TEM: Transmission electron microscopy
NTA: Nanoparticle tracking analysis
RIPA: Radioimmunoprecipitation assay
ALT: Alanine aminotransferase
AST: Aspartate aminotransferase
LDH: Lactate dehydrogenase
RT-qPCR: Quantitative real time polymerase chain reaction
ChIP: Chromatin immunoprecipitation
CLP: Cecal ligation and puncture
PLS-DA: Partial least square-discriminant analysis
DCFH-DA: 2′,7′-Dichlorfluorescein diacetate
NOX: NADPH oxidase
ROC: Receiver operating characteristic
AUC: Area under the curve
CKD: Chronic kidney disease
CCVD: Cardiovascular and cerebrovascular diseases
SOFA: Sequential organ failure assessment
