# Correction: Extracellular vesicles derived from CD4 ^+^ T cells carry DGKK to promote sepsis-induced lung injury by regulating oxidative stress and inflammation

**DOI:** 10.1186/s11658-024-00648-9

**Published:** 2024-10-28

**Authors:** Guo-wei Tu, Yi Zhang, Jie-fei Ma, Jun-yi Hou, Guang-wei Hao, Ying Su, Jing-chao Luo, Lulu Sheng, Luo Z

**Affiliations:** 1 Cardiac Intensive Care Center, Zhongshan Hospital, Fudan University, Shanghai, China; 2 Biomedical Research Center, Institute for Clinical Sciences, Zhongshan Hospital, Fudan University, Shanghai, China; 3 Department of Critical Care Medicine, Zhongshan Hospital (Xiamen), Fudan University, Xiamen, China; 4 Department of Emergency Medicine, Shanghai Jiao Tong University Affiliated Sixth People’s Hospital, Shanghai, China; 5 Shanghai Key Laboratory of Lung Inflammation and Injury, Shanghai, China; 6 Department of Critical Care Medicine, Shanghai Xuhui Central Hospital, ZhongshanXuhui Hospital, Fudan University, Shanghai, China

**Correction** **:** **Cellular & Molecular Biology Letters (2023) 28:24** 10.1186/s11658-023-00435-y

Following publication of the original article [1], the authors identified a similarity between the image labeled “CLP + shNC” in Fig. 6C of their article and an image in another publication from their hospital (PubMed: 34616481, Figure 5a). Upon careful investigation, the authors discovered that the image in question had been incorrectly labeled. The error occurred unintentionally due to the fact that two research teams from hospital, both studying acute lung injury, were working in the same laboratory. This resulted in an accidental mislabeling of the H&E histopathology slides because of an incorrect slide number in the shared platform.

The correct Fig. 6 is given below:

**Fig. 6:**
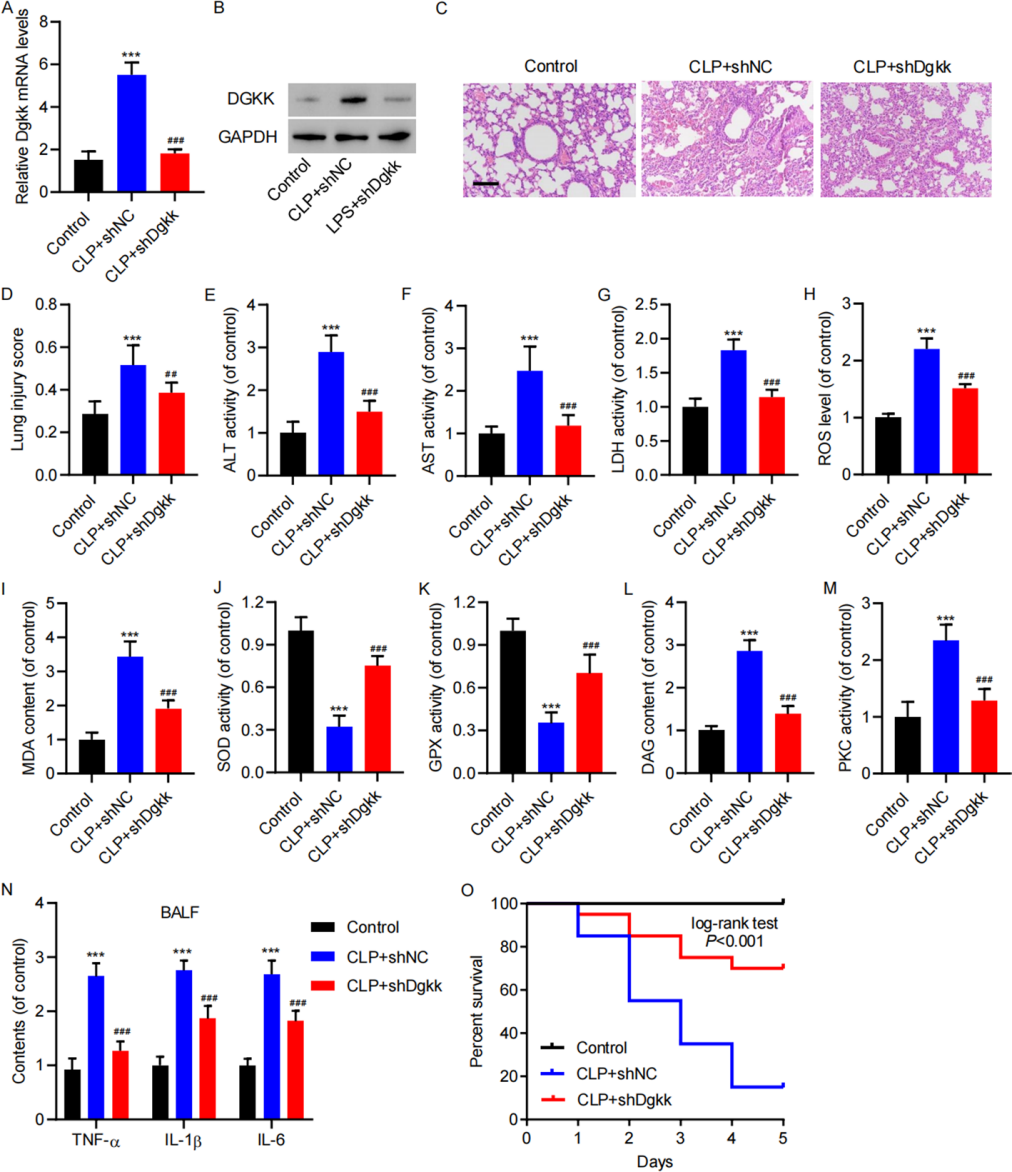
DGKK knockdown attenuated tissue damage, oxidative stress, and inflammation in mice with CLP-induced lung injury. Mice with CLP were injected with virus carrying plasmid expressing shRNA control (shNC) and shRNA targeting DGKK (shDgkk). The elevation of Dgkk abundance at the **A** mRNA and **B** protein levels in CLP model mice was restored by DGKK RNAi. **C** H and E staining indicated that the severe lung injury in CLP model mice was restored by Dgkk RNAi (scale bar, 100 μm). **D** The severity of histological injury and plasma levels of **E** ALT, **F** AST, and **G** LDH in CLP model mice was restored by DGKK RNAi. The elevation of **H** ROS levels and **I** MDA content in CLP model mice was restored by DGKK RNAi. The reduction of **J** SOD and **K** GPX activities in CLP model mice was restored by Dgkk RNAi. The elevation of **L** DAG content and **M** PKC activity in CLP model mice was restored by Dgkk RNAi. **N** The elevation of BALF content of TNF-α, IL-1β, and IL-6 in CLP model mice was restored by Dgkk RNAi. **O** The shortened survival in CLP model mice was restored by Dgkk RNAi. Data are presented as mean ± SD. ***P < 0.001 versus control. ^##^*P* < 0.01, ^###^*P* < 0.001 versus CLP + shNC

The authors re-examined the original samples and thoroughly reviewed the data, and they are confident that this mistake does not alter the overall conclusions of the article.
