# Supplementary material for: Extracellular vesicles derived from CD4+ T cells carry DGKK to promote sepsis-induced lung injury by regulating oxidative stress and inflammation
Source: Cell Mol Biol Lett. 2023 Mar 23;28:24. doi: 10.1186/s11658-023-00435-y (PMC10035494; doi:10.1186/s11658-023-00435-y)
Supplement: Supplementary file 1 — Additional file 1: Figure S1. Characterization of EVs isolated from the serum of patients with sepsis-induced lung injury and healthy control subjects. A TEM observation (scale bar, 200 nm). B Western blot analysis of EV markers. C Diameter distribution of EVs by NTA. Figure S2. LPS promoted DGKK expression via the NF-κB pathway in human CD4+ T cells. Elevation of DGKK expression at the A mRNA and B protein levels in CD4+ T cells isolated from healthy subjects treated with 10 μg/mL LPS at various treatment durations. C Treatment of QNZ strongly suppressed the upregulation of TLR4 and higher nuclear/cytoplasmic distribution of NF-κB p65 in LPS-treated CD4+ T cells, as shown by western blot. D The elevation of DGKK expression at the mRNA and protein levels in human CD4+ T cells treated with LPS was restored by QNZ. E Luciferase reporter assay showed that the WT promoter of the DGKK gene was activated by LPS treatment, which could be restored by QNZ. However, the mutant promoter of the DGKK gene could not be activated by LPS. F The NF-κB binding site in the DGKK promoter was predicted using JASPAR. G ChIP–qPCR showed that the elevation of NF-κB binding to the DGKK promoter by LPS treatment was restored by QNZ. ***P < 0.001 versus 0 h or control. ###P < 0.001 versus LPS Figure S3. Characterization of EVs isolated from cultured human CD4+ T cells treated with or without 10 μg/mL LPS. A TEM observation (scale bar, 200 nm). B Western blot analysis of EV markers and DGKK. C Diameter distribution of EVs by NTA. D Laser scanning confocal microscope analysis of EV uptake by A549 cells (scale bar, 50 μm). Figure S4. Toxic effects of CD4+ T-cell-derived EVs on oxidative stress and inflammation in mice. Mice were treated with CLP, SSE, SE, LTE, or TE. A H and E staining (scale bar, 100 μm). B Severity of histological injury. Plasma levels of C ALT, D AST, E LDH, and F ROS level, G MDA content, H SOD activity, I GPX activity, and I BALF content of TNF-α, IL-1β, and IL-6 in lung ti [file 11658_2023_435_MOESM1_ESM.docx]

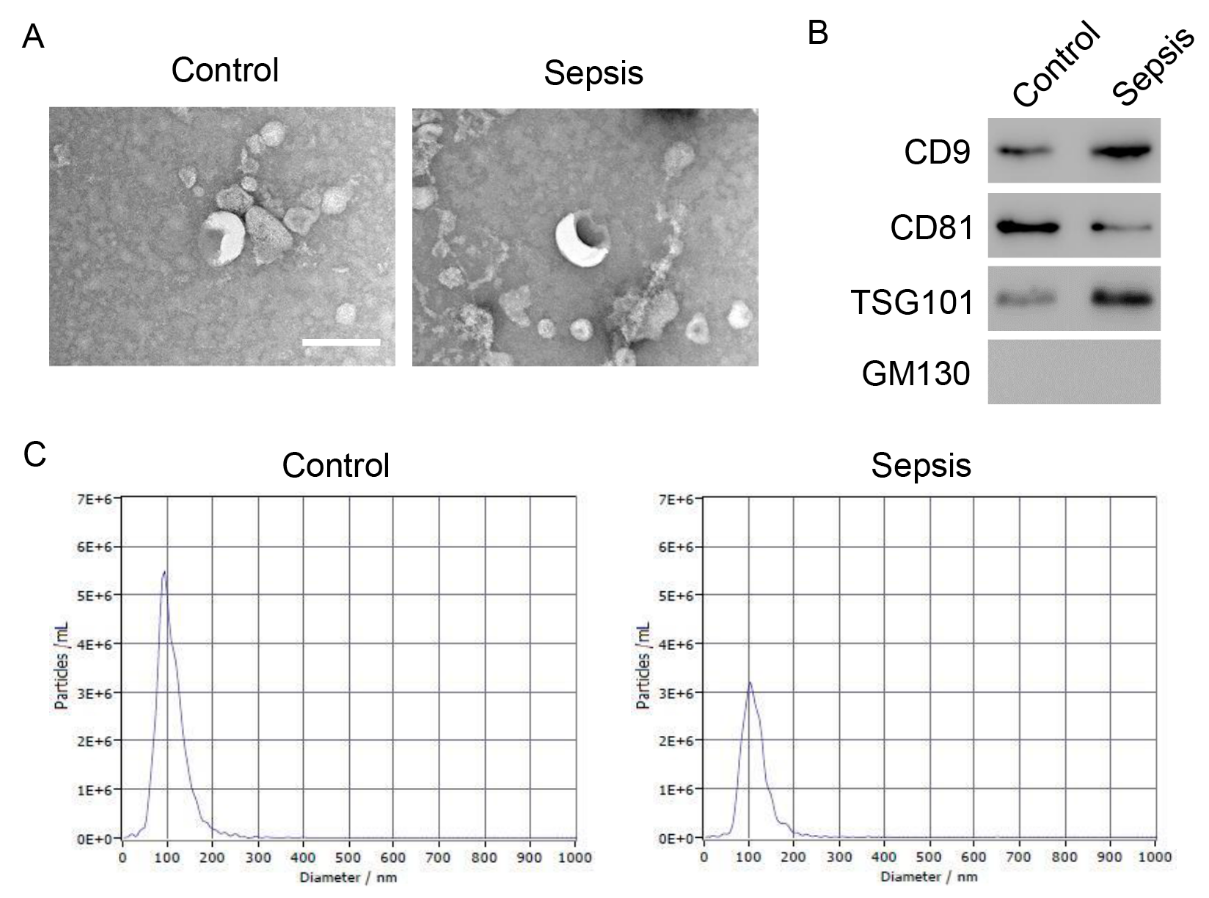


**Figure S1. Characterization of EVs isolated from the serum of patients with sepsis-induced lung injury and healthy control subjects.** (A) TEM observation (scale bar, 200 nm). (B) Western blot analysis of EV markers. (C) Diameter distribution of EVs by NTA.


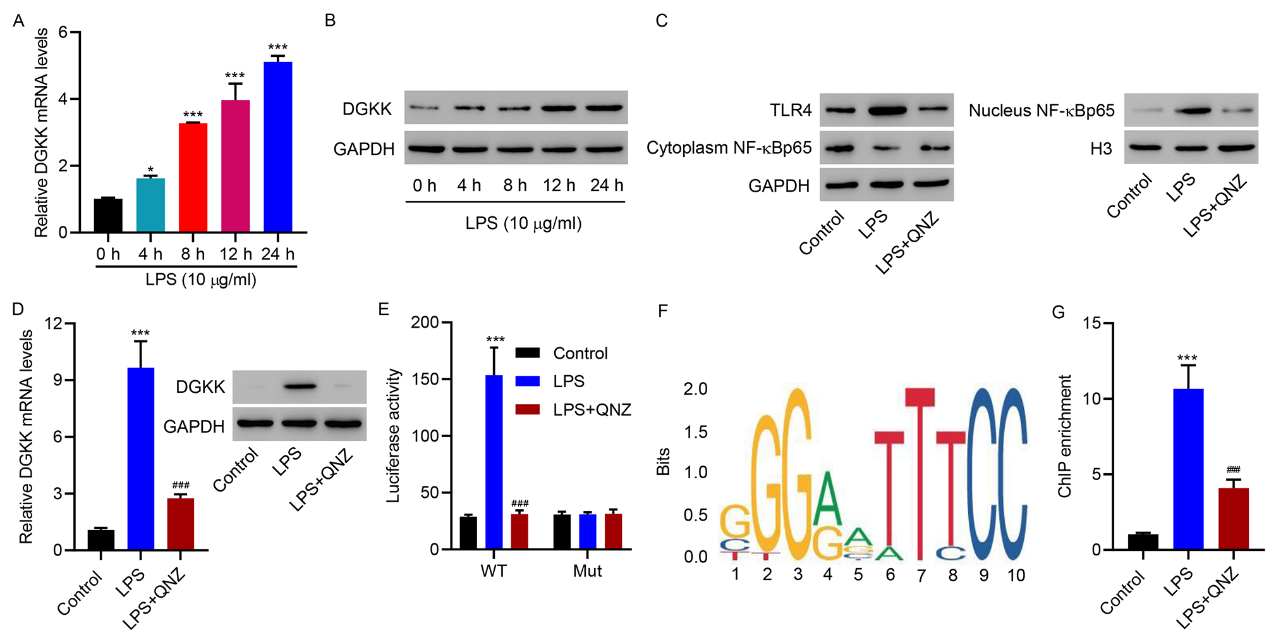


**Figure S2. LPS promoted DGKK expression via the NF-κB pathway in human CD4^+^ T cells.** Elevation of DGKK expression at the (A) mRNA and (B) protein levels in CD4^+^ T cells isolated from healthy subjects treated with 10 μg/mL LPS at various treatment durations. (C) Treatment of QNZ strongly suppressed the upregulation of TLR4 and higher nuclear/cytoplasmic distribution of NF-κB p65 in LPS-treated CD4^+^ T cells, as shown by Western blot. (D) The elevation of DGKK expression at the mRNA and protein levels in human CD4^+^ T cells treated with LPS was restored by QNZ. (E) Luciferase reporter assay showed that the WT promoter of the DGKK gene was activated by LPS treatment, which could be restored by QNZ. However, the mutant promoter of the DGKK gene could not be activated by LPS. (F) The NF-κB binding site in the DGKK promoter was predicted using JASPAR. (G) ChIP–qPCR showed that the elevation of NF-κB binding to the DGKK promoter by LPS treatment was restored by QNZ. ***P < 0.001 vs. 0 h or control. ^###^P < 0.001 vs. LPS.


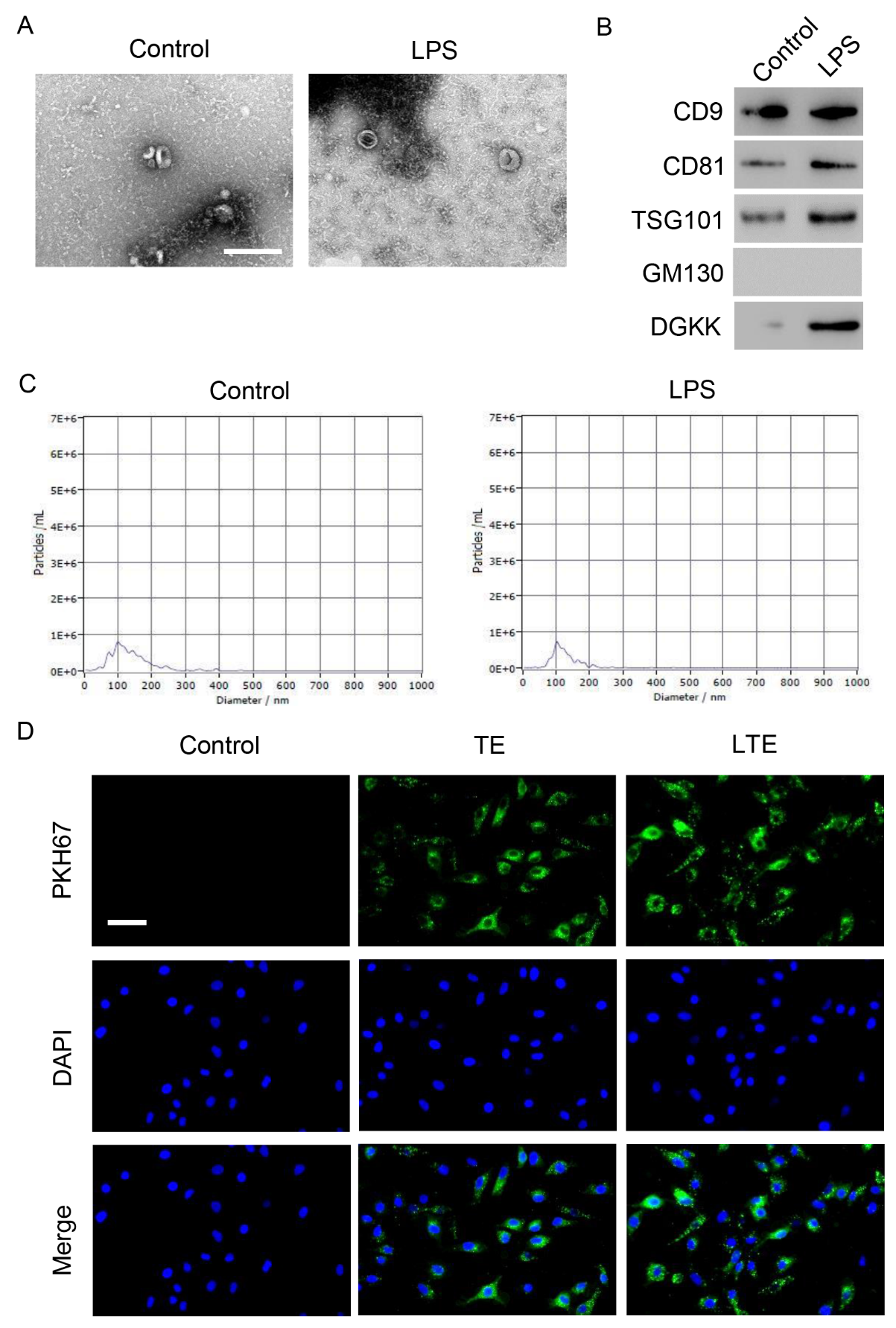


**Figure S3. Characterization of EVs isolated from cultured human CD4^+^ T cells treated with or without 10 μg/mL LPS.** (A) TEM observation (scale bar, 200 nm). (B) Western blot analysis of EV markers and DGKK. (C) Diameter distribution of EVs by NTA. (D) Laser scanning confocal microscope analysis of EV uptake by A549 cells (scale bar, 50 μm).


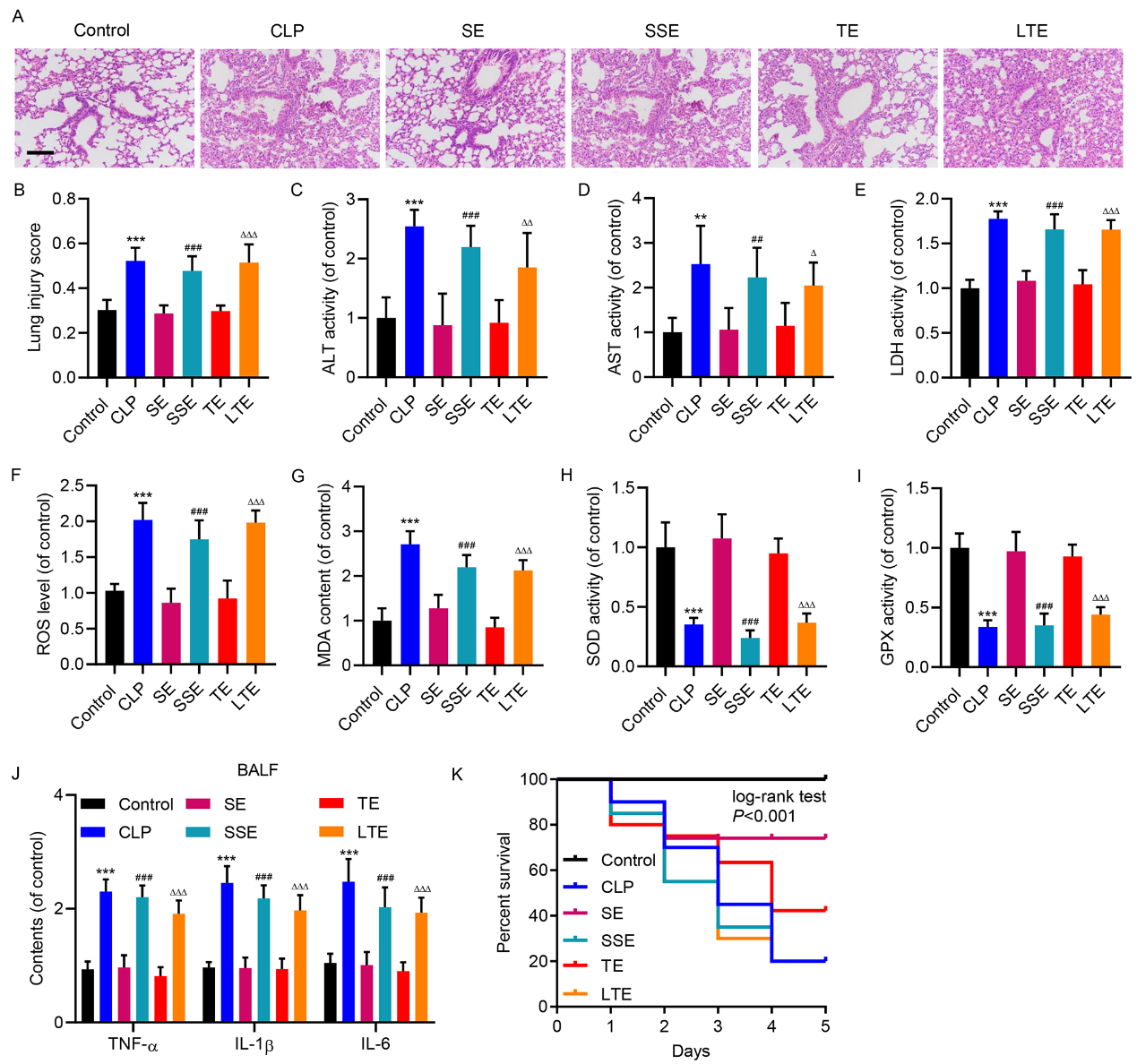


**Figure S4. Toxic effects of CD4^+^ T cell-derived EVs on oxidative stress and inflammation in mice.** Mice were treated with CLP, SSE, SE, LTE, or TE. (A) H&E staining (scale bar, 100 μm). (B) Severity of histological injury. Plasma levels of (C) ALT, (D) AST, (E) LDH, and (F) ROS level, (G) MDA content, (H) SOD activity, (I) GPX activity) and (J) BALF content of TNF-α, IL-1β, and IL-6 in lung tissues of mice. (K) The survival rate of mice was monitored within 5 days, showing the shortened survival with EV treatment. Data are presented as mean ± SD. ***P < 0.001 vs. control. ^##^P < 0.01, ^###^P < 0.001 vs. SE. ^Δ^P < 0.05, ^ΔΔ^P < 0.01, ^ΔΔΔ^P < 0.001 vs. TE.


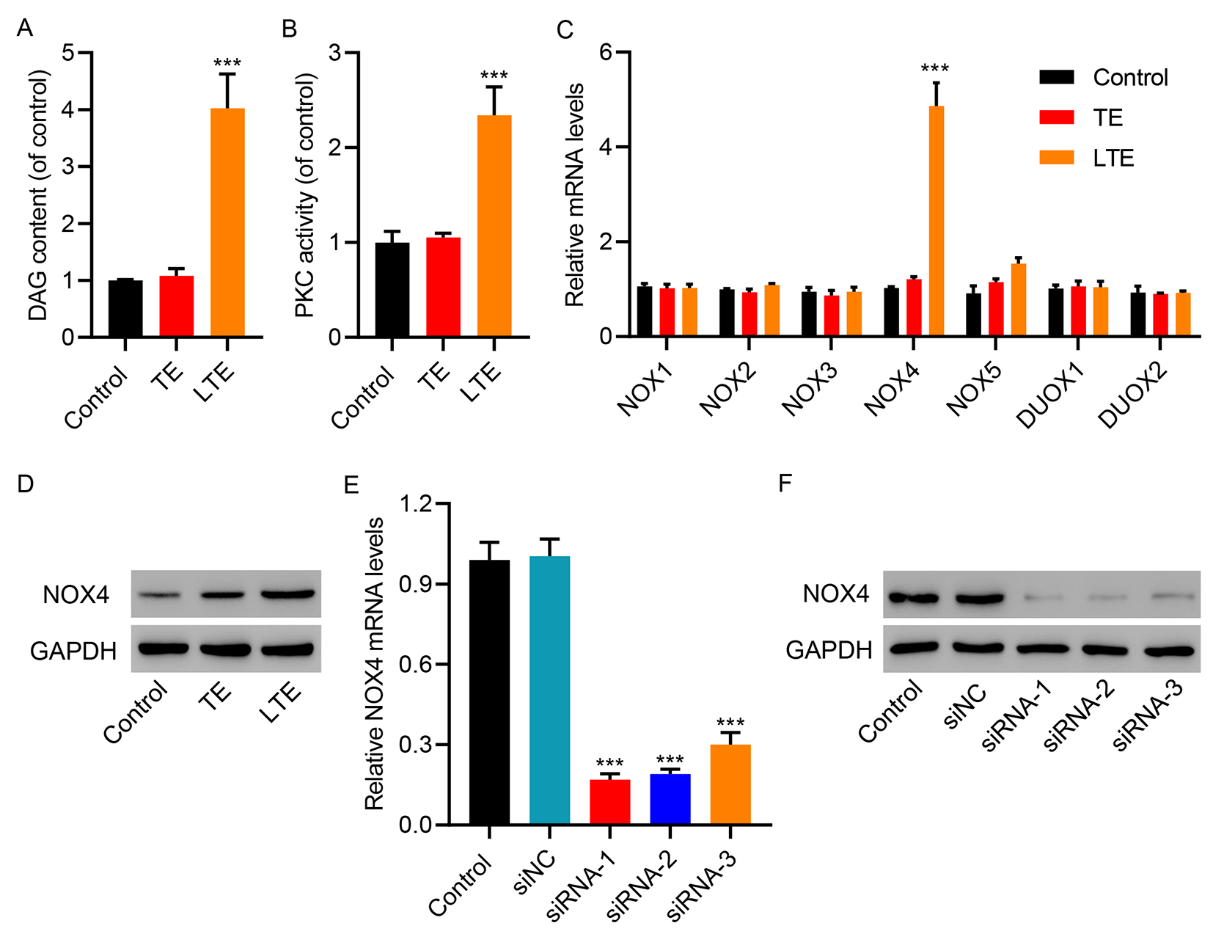


**Figure S5. Activation of the DAG/PKC/NOX4 signaling pathway by T cell EVs.** A549 cells were treated with EVs isolated from CD4^+^ T cells isolated from healthy subjects treated with (LTE) or without (TE) 10 μg/mL LPS. (A) DAG content and (B) PKC activity were increased by LTE. (C) The mRNA expression of NOX4 in A549 cells was upregulated by LTE, but not NOX1, NOX2, NOX3, NOX5, DUOX1, and DUOX2. (D) The protein level of NOX4 in A549 cells was upregulated by LTE. The expression of NOX4 in A549 cells was significantly suppressed by NOX4 siRNA at the (E) mRNA and (F) protein levels. Data are presented as mean ± SD. ***P < 0.001 vs. control or siNC.


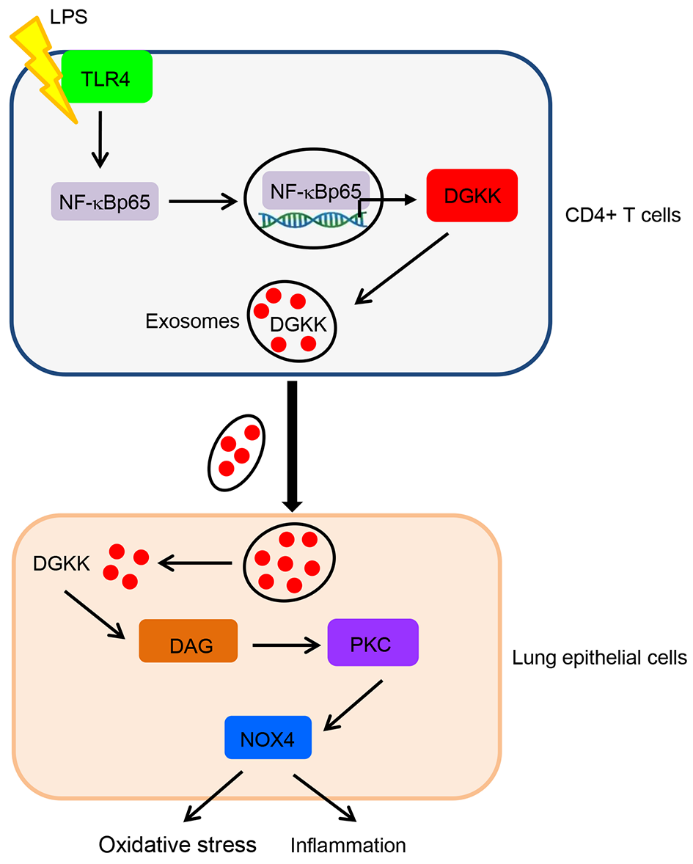


**Figure S6. Schematic representation of the regulation of oxidative stress and inflammation in lung injury by EVs from CD4^+^ T cells via the DGKK/DAG/PKC/NOX4 pathway.**
